# CircXRN2 suppresses tumor progression driven by histone lactylation through activating the Hippo pathway in human bladder cancer

**DOI:** 10.1186/s12943-023-01856-1

**Published:** 2023-09-08

**Authors:** Bo Xie, Juntao Lin, Xianwu Chen, Xuejian Zhou, Yan Zhang, Mengjing Fan, Jiayong Xiang, Ning He, Zhenghui Hu, Feifan Wang

**Affiliations:** 1 Department of Urology, The First Affiliated Hospital, Zhejiang University School of Medicine, Hangzhou, Zhejiang 310003 R.P. China; 2 Department of Pathology, Sir Run Run Shaw Hospital, Zhejiang University School of Medicine, Hangzhou, Zhejiang 310016 R.P. China

**Keywords:** Bladder cancer, circXRN2, Hippo pathway, Histone lactylation, Tumor progression

## Abstract

**Background:**

Bladder cancer (BCa) is the fourth most common malignant tumor with a poor prognosis worldwide. Further exploration and research are needed to unmask the underlying roles and molecular mechanisms of circular RNAs. In the current study, our findings showed that circXRN2 suppresses tumor progression driven by histone lactylation by activating the Hippo pathway in human bladder cancer.

**Methods:**

RNA immunoprecipitation (RIP) followed by circRNA sequencing confirmed circXRN2 as the research object. Overexpression of circXRN2 and knockdown of TAZ/YAP further verified the biological functions in T24 and TCCSUP cells. RIP, immunoprecipitation and coimmunoprecipitation were used to elucidate the interaction between circXRN2 and LATS1. A Seahorse metabolic analyzer was used to determine the glycolytic rate. Cleavage under targets and Tagmentation (CUT&Tag) and chromatin immunoprecipitation (ChIP) were employed to ensure the regulatory roles of H3K18 lactylation in the transcriptional activity of LCN2.

**Results:**

CircXRN2 is aberrantly downregulated in bladder cancer tissues and cell lines. CircXRN2 inhibits the proliferation and migration of tumor cells both in vitro and in vivo. In addition, circXRN2 serves as a negative regulator of glycolysis and lactate production. Mechanistically, circXRN2 prevents LATS1 from SPOP-mediated degradation by binding to the SPOP degron and then activates the Hippo signaling pathway to exert various biological functions. The circXRN2-Hippo pathway regulatory axis further modulates tumor progression by inhibiting H3K18 lactylation and LCN2 expression in human bladder cancer.

**Conclusions:**

CircXRN2 suppresses tumor progression driven by H3K18 lactylation by activating the Hippo signaling pathway in human bladder cancer. Our results indicated novel therapeutic targets and provided promising strategies for clinical intervention in human bladder cancer.

**Supplementary Information:**

The online version contains supplementary material available at 10.1186/s12943-023-01856-1.

## Background

Bladder cancer is the fourth most common malignant tumor and the 13^th^ leading cause of death in cancer patients worldwide [1, 2]. In addition, bladder cancer is the most common cancer in the urinary system in China [3]. Approximately 90% of bladder cancers are uroepithelial cancers. According to the depth of invasion, BCa can be classified into nonmuscle-invasive bladder cancer (NMIBC) and muscle-invasive bladder cancer (MIBC) [4]. A total of 19.8%-54% of high-risk NMIBC patients will progress to MIBC [5]. The overall 5-year survival rate of MIBC is approximately 60%-70%. About 10% of new-onset MIBC is metastasized, and the 5-year survival rate is 5%-30%. Localized MIBC is feasible for radical cystectomy and pelvic lymph node dissection, but approximately half of these patients will have distant metastasis and recurrence after surgery [1, 6]. Therefore, more in-depth investigations into the tumorigenesis and progression of bladder cancer are of great significance.

With the updating and advancement of high-throughput sequencing and molecular biological technologies, noncoding RNAs have exerted emerging roles in various physiological and pathological processes. CircRNA is a profound mystical kind of long noncoding RNA. Most circRNAs consist of exons from existing protein-coding genes through noncanonical back-splicing [7]. Because of their covalent loop structure, circRNAs can tolerate degradation by exonucleases and are more stable than linear RNAs [8]. Emerging literature has reported that circRNAs regulate the occurrence and development of multiple human diseases, especially cancers [9–11]. To date, it has been elucidated that circRNAs can serve as microRNA sponges or decoys [12, 13], modulate the expression levels of specific proteins [14, 15], and serve as scaffolds accelerating interactions between proteins by colocalization [16, 17]. Our previous studies have indicated that circRNAs participate in bladder cancer progression by acting as miRNA sponges [18, 19]. However, more thorough research on circRNAs modulating the tumorigenesis and development of bladder cancer is of great significance.

The Hippo pathway is a highly conserved modulating system controlling organ development, regeneration of tissue, cell proliferation and immune regulation [20]. Upstream signaling transmission of the Hippo pathway is mainly mediated by phosphorylation. Phosphorylated TAZ/YAP is retained in the cytoplasm and eventually degraded. LATS1, a core factor in the kinase cascade of the Hippo pathway, plays a vital role during this process. LATS1 phosphorylates TAZ/YAP, increasing the retention rate of TAZ/YAP in the cytoplasm and thus suppressing the transcriptional activity of TAZ/YAP [21]. Notably, emerging evidence supports the aberrant status of the Hippo pathway in human cancer, promoting tumorigenesis, metastatic progression, and metabolic shift [22–25].

Histone lactylation was newly discovered by Zhang et al. in 2019. This brand-new epigenetic modification relies on lactate produced by intracellular metabolism and regulates cell biological functions by activating downstream gene transcription and expression [26]. Aerobic glycolysis, also termed the Warburg effect, is one of the most characteristic features of tumor cells and refers to the preferential production of energy through glycolysis rather than oxidative phosphorylation even under normoxia [27]. Therefore, tumor cells will produce and accumulate more lactate than normal cells, which makes the functions of histone lactylation worth exploring in tumors. However, the specific roles of histone lactylation in human bladder cancer remain unclear.

Here, we discovered, for the first time, that circXRN2 activates the Hippo pathway by stabilizing LATS1, which in turn suppresses H3K18 lactylation-driven tumor progression in human bladder cancer. Our findings provide an original molecular mechanism of circRNA and expand our understanding of the Hippo pathway and histone lactylation in bladder cancer.

## Materials and methods

### Ethical approval

The use of clinical samples with patient consent (Approval Number: IIT20220447B) and the xenograft tumorigenesis model and lung-metastatic model (Approval Number: 20221595) in this study were approved by the Ethical Committee, The First Affiliated Hospital, Zhejiang University School of Medicine, and the operations and experimental protocols were performed according to the laboratory guidelines of the NIH. Detailed information on the surgical samples is listed in Supplementary file 1.

### RNA immunoprecipitation (RIP)

An RNA Immunoprecipitation Kit (Geneseed, Guangzhou) was used for the RIP assay according to the manufacturer’s instructions. In brief, 100 μL of cell lysates was set aside for input control. To obtain the antibody-bead complex, pretreated magnetic Protein A + G beads were coincubated with specific antibodies and IgG (5 μg) at 4 °C and rotated for 120 min at 10 rpm. After the reaction was completed, the supernatant was discarded on the magnetic frame. RNA complexes bound to beads were washed, and RNA was extracted for subsequent assays.

### CUT&Tag

The CUT&Tag assay was performed using the Hyperactive In-Situ ChIP Library Prep Kit for Illumina according to the manufacturer’s instructions. Briefly, prepared concanavalin A-coated magnetic beads (ConA beads) were added to resuspended cells and incubated at room temperature to bind cells. The nonionic detergent digitonin was used to permeate the cell membrane. Then, H3K18la antibody (PTM-1427RM, PTM BIO, Hangzhou, China), secondary antibody and Hyperactive pA-Tn5 Transposase were incubated with the cells that were bound by ConA beads. Therefore, the hyperactive pA-Tn5 transposase can exactly cut off the DNA fragments that were bound with the target protein. In addition, the cut DNA fragments can be ligated with P5 and P7 adaptors by Tn5 transposase, and the libraries were amplified by PCR with the P5 and P7 primers. The purified PCR products were evaluated using the Agilent 2100 Bioanalyzer (Agilent Technologies, Santa Clara, CA, USA). Finally, these libraries were sequenced on the Illumina NovaSeq6000 platform, and 150 bp paired-end reads were generated for the following analysis. The results are presented in Supplementary files 6 and 7.

### RNA fluorescence in situ hybridization (FISH)

The Cy3-labeled circXRN2 probe was synthesized and obtained from RiboBio (Guangzhou, PR China). Later, a FISH Detection Kit (RiboBio, PR China) was employed according to the manual. Cell nuclei were stained with DAPI. The results were captured by a microscope (Olympus, Tokyo, Japan). The details of the probe used in this study are listed in Supplementary file 3.

### Seahorse metabolic analyzer glycolytic rate assay

Agilent Seahorse XFe24, a noninvasive and real-time analyzer of glycolysis in living cells, was used to evaluate the glycolytic rate of different cells in this research. The rate of glycolysis can be reflected by the extracellular acidification rate (ECAR). To eliminate the influence of mitochondrial respiration, the extracellular acidification caused by mitochondrial respiration is calculated through the oxygen consumption rate (OCR). Briefly, equal numbers of tumor cells (5 × 10^4^) were seeded into 24-well plates. The next day, the cells were rinsed with Seahorse detection buffer. Then, the analyzer injected Rot/AA and 2-DG (2-deoxy-glucose) automatically. The glycolytic proton efflux rate (glycoPER), basal glycolysis rate and compensatory glycolysis rate can be calculated to reflect the real-time glycolytic status of cells by the analyzer.

### Glycolytic process evaluation

To evaluate the status of glycolysis, 2-NBDG and Glucose Uptake Colorimetric Assay Kits (Biovision, USA) were used to determine glucose uptake ability, and the production of lactate was measured by lactate colorimetric assay kits (Biovision, USA). All procedures were performed according to the manual.

### Cell lines and culture

All cell lines cultured in this study were obtained from the Chinese Academy of Sciences. In detail, RPMI 1640 medium supplemented with 10% fetal bovine serum (FBS) and 1% Penicillin‒Streptomycin Solution (P/S) was prepared for culturing 5637, T24 and EJ; F-12K medium supplemented with 10% FBS and 1% P/S was prepared for culturing SV-HUC-1; MEM medium supplemented with 10% FBS and 1% P/S was prepared for TCCSUP; DMEM supplemented with 10% FBS and 1% P/S was prepared for UM-UC-3. The culture conditions were 37 °C and 5% CO_2_.

### RNA extraction and quantitative real-time PCR analysis

Total RNA was extracted by NucleoZOL® RNA Isolation Reagent (MACHEREY–NAGEL, Germany). After reverse transcription by using the PrimeScript™ RT Reagent Kit (Perfect Real Time), polymerase chain reaction was conducted by an Applied Biosystems QUANT5 Studio PCR system. GAPDH was used as an endogenous control. The amplification conditions were as follows: a. denaturation at 95 °C for 10 s; b. annealing at 72 °C for 20 s; and c. extension at 60 °C for 20 s for 40 cycles. The Ct values were collated according to the results, and the 2^−ΔΔCt^ method was used to calculate the relative expression levels of target genes. The primers used in this study are listed in Supplementary file 3.

### SiRNA, plasmid and lentivirus

SiRNAs were obtained from TranSheepBio (Shanghai, China). We transfected siRNAs into cells with Lipofectamine RNAiMAX (Invitrogen). To overexpress circXRN2 in bladder cancer cells, Genomeditech (Shanghai, China) designed and obtained cDNA containing full-length circXRN2 and embedded it into the pcD-ciR vector. Then, the plasmids were transfected into cells via Lipofectamine 3000 (Invitrogen). For lentivirus-related experiments, we treated cells with puromycin for one week to ensure the efficiency of transfection. The sequences of siRNAs in this study are listed in Supplementary file 3.

### Chromatin immunoprecipitation (ChIP)

To investigate the interaction between H3K18la and the promoter region of LCN2, a SimpleChIP® Plus Enzymatic Chromatin IP Kit (CST, #9004, USA) was used according to the manufacturer’s procedure in our study.

### Protein immunoprecipitation (IP)

The IP experiment was performed using the Absin Co-IP kit (Absin, ab955, China) according to the manufacturer’s instructions. Briefly, a total of 1 × 10^7^ cells were collected and washed with PBS. After 15 min of lysis, the supernatant was incubated with specific antibodies at 4 °C for 12 h with rotation. The next day, 5 μL of Protein A and 5 μL of Protein G were added, incubated with rotation for 2 h at 4 °C, and then centrifuged at 12,000 × g for 1 min. Finally, the pellet was retained for later use.

### CCK-8 assay

To evaluate cell viability, we employed Cell Counting Kit-8 (APEBIO, USA) according to the manufacturer’s instructions. Briefly, 1 × 10^4^ pretreated cells were seeded into 96-well plates after counting, and the volume of medium added to each well was 100 μL. The next day, 10 μL CCK8 solution was added to each well, and the cells were incubated for 2 h in a 37 °C incubator. Finally, absorbance (450 nm) was measured by a SpectraMax i3x reader. Before relative cell viability was calculated, the values of the blank control well were subtracted.

### Colony formation assay

A total of 2000 cells were cultured in 12-well plates for colony formation. After one week of incubation, the cells were fixed with 4% paraformaldehyde and stained with crystal violet. The colonies were photographed and counted.

### Transwell migratory experiment

To evaluate cell migratory ability, equal numbers (3 × 10^4^) of T24 cells were seeded into the upper chamber and incubated overnight. Subsequently, 500 μL complete medium supplemented with 10% FBS was added into the lower chamber of the Transwell insert to promote cell migration. After another 24-h incubation, cells migrating through the membrane of Transwell inserts were stained with crystal violet and photographed by microscopy (100 ×).

### Wound healing assay

We used an Ibidi culture insert (Ibidi, Germany) to evaluate the migratory ability of different cells. Briefly, bladder cancer cells were seeded into cultured plates according to the manufacturer’s protocol and incubated overnight. The next day, the inserts were removed, and serum-free medium was added. After 36 h, bright field images were acquired with a microscope. ImageJ was used to calculate the migratory rate.

### Analysis of apoptosis

The apoptotic rate was determined by an Apoptosis Detection Kit (BD, USA). Annexin V-FITC binds to outward phosphatidylserine in the early stage of apoptosis. In addition, necrotic or late apoptotic cells can be stained by PI. Briefly, cells were harvested and stained with Annexin V-FITC and PI at room temperature. The apoptotic rate was determined and analyzed by BD FACS Calibur (BD, USA) and FlowJo software.

### Western blot

RIPA buffer (Beyotime, Shanghai) was used to extract total protein from cells. Then, we measured the concentration of protein with a BCA assay kit (Beyotime, Shanghai). Furthermore, samples were separated by SDS‒PAGE and transferred to polyvinylidene fluoride (PVDF) membranes. Primary antibodies against specific genes were diluted in the NCM Universal Antibody Diluent (WB500D, NCM Biotech, China) and incubated with PVDF membranes overnight at 4 °C. The next day, the membranes were incubated with secondary antibodies at room temperature for 2 h. The chemiluminescence method was used to determine the relative expression levels of different genes. Antibody against H3K18la were purchased from PTM BIO (PTM-1406RM, Hangzhou, China).

### Immunofluorescence assay

Cells were fixed with 4% paraformaldehyde and permeabilized with 0.1% Triton-X. Later, 2.5% BSA solution was used for blocking. After incubation with primary antibodies and secondary antibodies, images were captured by a microscope (Nikon, Japan). The nuclei were stained with DAPI.

### Subcutaneous tumorigenesis model

T24 cells (6 × 10^6^) were suspended in PBS and injected subcutaneously into nude mice (4 weeks old). The parameters of subcutaneous tumors were recorded for 7 days. Of note, the volume of the tumor was speculated by the formula below: tumor volume = 0.5 × length × width^2^ (mm^3^). After 35 days, tumors were harvested from sacrificed mice and weighed. We took care of the experimental animals in accordance with the guidelines of the Laboratory Animal Center, Zhejiang University.

### Tail vein-lung metastasis model

T24 cells (5 × 10^6^) were suspended in PBS and injected via the tail vein into 6-week-old nude mice. Fifty days later, the mice were sacrificed, and the lung tissues were dissected. Tissues were fixed for H&E staining. We took care of the experimental animals in accordance with the guidelines of the Laboratory Animal Center, Zhejiang University.

### Statistical analysis

Differences between groups were analyzed by one-way or two-way ANOVA in SPSS software, and the data in this study are presented as the mean ± standard deviation (SD). A *P* value < 0.05 was regarded as statistically significant.

## Results

### LATS1 is downregulated in bladder cancer and interacts with circXRN2

**Fig. 1:**
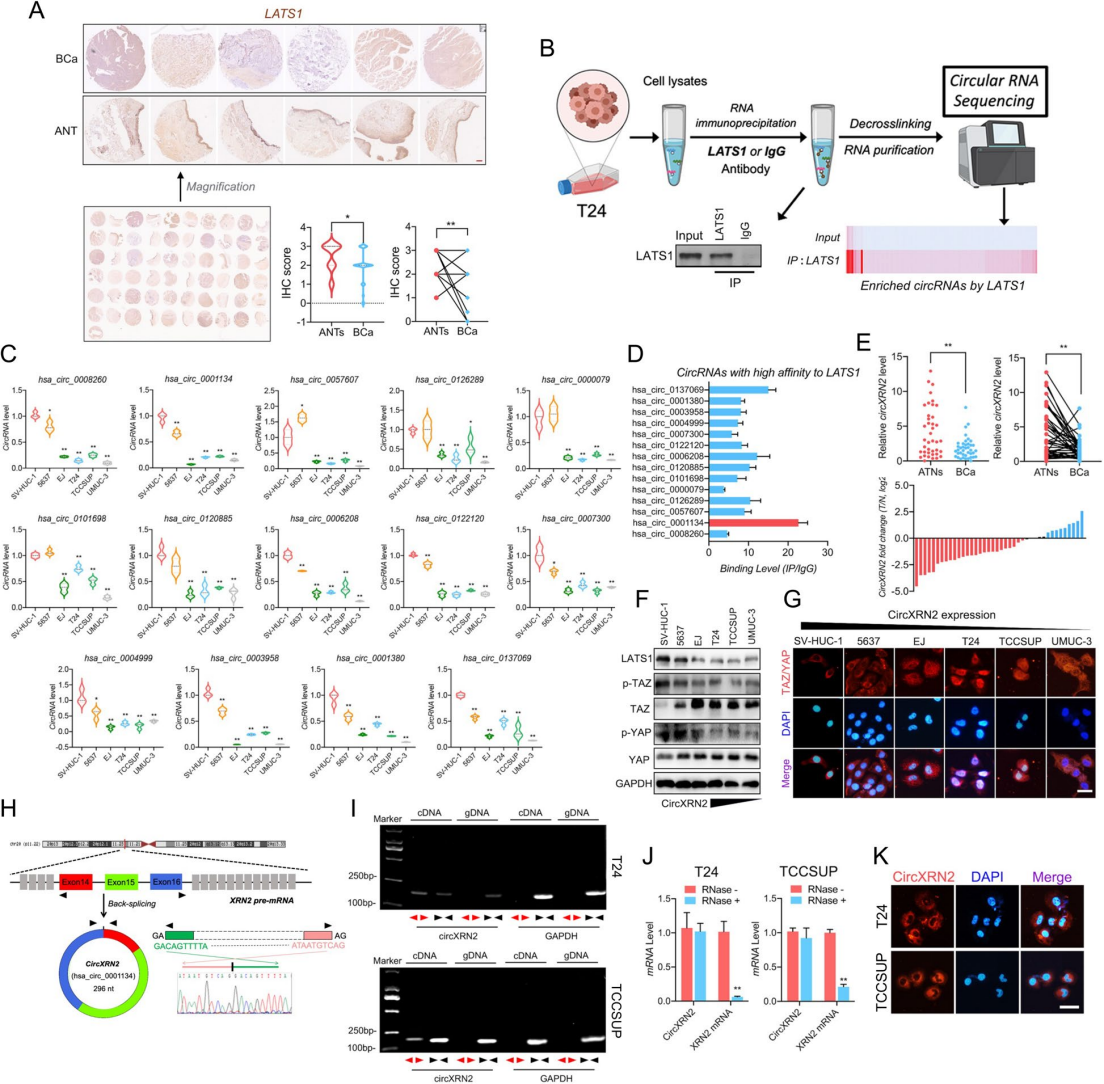
LATS1 is downregulated and interacts with circXRN2 in bladder cancer. **a** LATS1 was downregulated in surgical tissue samples (*n* = 30). Scale bar: 200 μm. **b** RIP assay followed by RNA sequencing revealed that LATS1 protein could interact with numerous circRNAs. **c** CircRNAs binding with LATS1 were validated by qRT‒PCR in different cell lines (SV-HUC-1, 5637, EJ, T24, TCCSUP and UM-UC-3). **d** The affinities of dysregulated circRNAs to the LATS1 protein were evaluated by RIP assay, and the results showed that circXRN2 had the highest affinity for LATS1. **e** Total RNA was extracted from clinical specimens of our own cohort (*n* = 40). Dysregulation of circXRN2 in surgical tissue samples was confirmed by qRT‒PCR. **f** The expression levels of LATS1, p-TAZ, TAZ, p-YAP and YAP in cell lines were determined by western blotting. **g** Immunofluorescence indicated the localization of TAZ/YAP in cell lines with various abundances of circXRN2. Scale bar: 50 μm. **h** The biological structure of circXRN2 is represented by a graphic illustration, and the back-splicing site was verified by PCR and Sanger sequencing. **i** CircXRN2 was only amplified by divergent primers in cDNA but not in gDNA of T24 and TCCSUP cells. GAPDH was used as a negative control. **j** Cells were incubated in the absence or presence of RNase R, and the expression levels of circXRN2 and linear XRN2 were determined by qRT‒PCR. **k** RNA FISH indicated that CircXRN2 was mainly located in the cytoplasm. The nuclei were stained with DAPI, while circXRN2 was red. Scale bar: 50 μm. All the data are presented as the mean ± standard deviation (*n* = 3). *P < 0.05, **P < 0.01, compared with the control group

LATS1 is a vital promoting molecule in the Hippo signaling pathway, playing a significant role in various malignancies [28, 29]. As shown in Fig. 1a, tissue microarrays and clinical samples indicated that LATS1 was aberrantly downregulated in bladder cancer tissues compared with adjacent normal tissues. By performing RNA immunoprecipitation (RIP) together with high-throughput sequencing, we obtained a database of circRNAs that potentially interact with the LATS1 protein (Fig. 1b and Supplementary File 2). To further discover the specific circRNAs that may participate in tumorigenesis and progression, we verified the expression level of circRNAs from the database in immortalized human normal urothelium cell (SV-HUC-1) and bladder cancer cell lines (5637, T24, EJ, TCCSUP and UM-UC-3). As a result we identified 14 circRNAs with remarkably low expression levels in bladder cancer cell lines (Fig. 1c). Furthermore, we used a RIP assay to validate the affinity between LATS1 and the circRNAs mentioned above, among which circXRN2 (hsa_circ_0001134) had the highest binding efficiency and affinity for the LATS1 protein (Fig. 1d). In addition, by qRT‒PCR, we verified the dysregulation of circXRN2 in clinical tumor tissues (Fig. 1e). Notably, the expression level of circXRN2 in tumor cell lines was associated with the activation of key Hippo pathway molecules, TAZ and YAP, to some extent (Fig. 1f and g). These findings suggested that circXRN2 might be involved in the modulation of LATS1 and the Hippo signaling pathway.

### CircXRN2 inhibits the proliferation and migration of bladder cancer cells in vitro and in vivo

CircRNAs are long noncoding RNAs featuring a covalently closed loop structure that protects them from degradation via exonucleases. Most of them are believed to consist of exons derived from existing protein-coding genes. CircXRN2 was formed by exon 14, exon 15 and exon 16 of the XRN2 gene located on chromosome 28 through back-splicing and consisted of 296 bp. According to Sanger sequencing, we confirmed the conjunctive site sequence of circXRN2 (Fig. 1h). Then, we performed qRT‒PCR by using divergent primers and convergent primers. The results of Fig. 1i demonstrated that circXRN2 could only be amplified by divergent primers in cDNA, which confirmed the loop structure of circXRN2 as opposed to a linear structure. Compared with linear RNAs, circRNA is more stable in the endogenous environment, so it is not easily degradable. Because of this property, we treated the samples with RNase R and found that circXRN2 could tolerate digestion by RNase R, while linear XRN2 mRNA was almost completely degraded (Fig. 1j). Last, RNA FISH indicated that circXRN2 was mainly localized in the cytoplasm in bladder cancer cells (Fig. 1k).

**Fig. 2:**
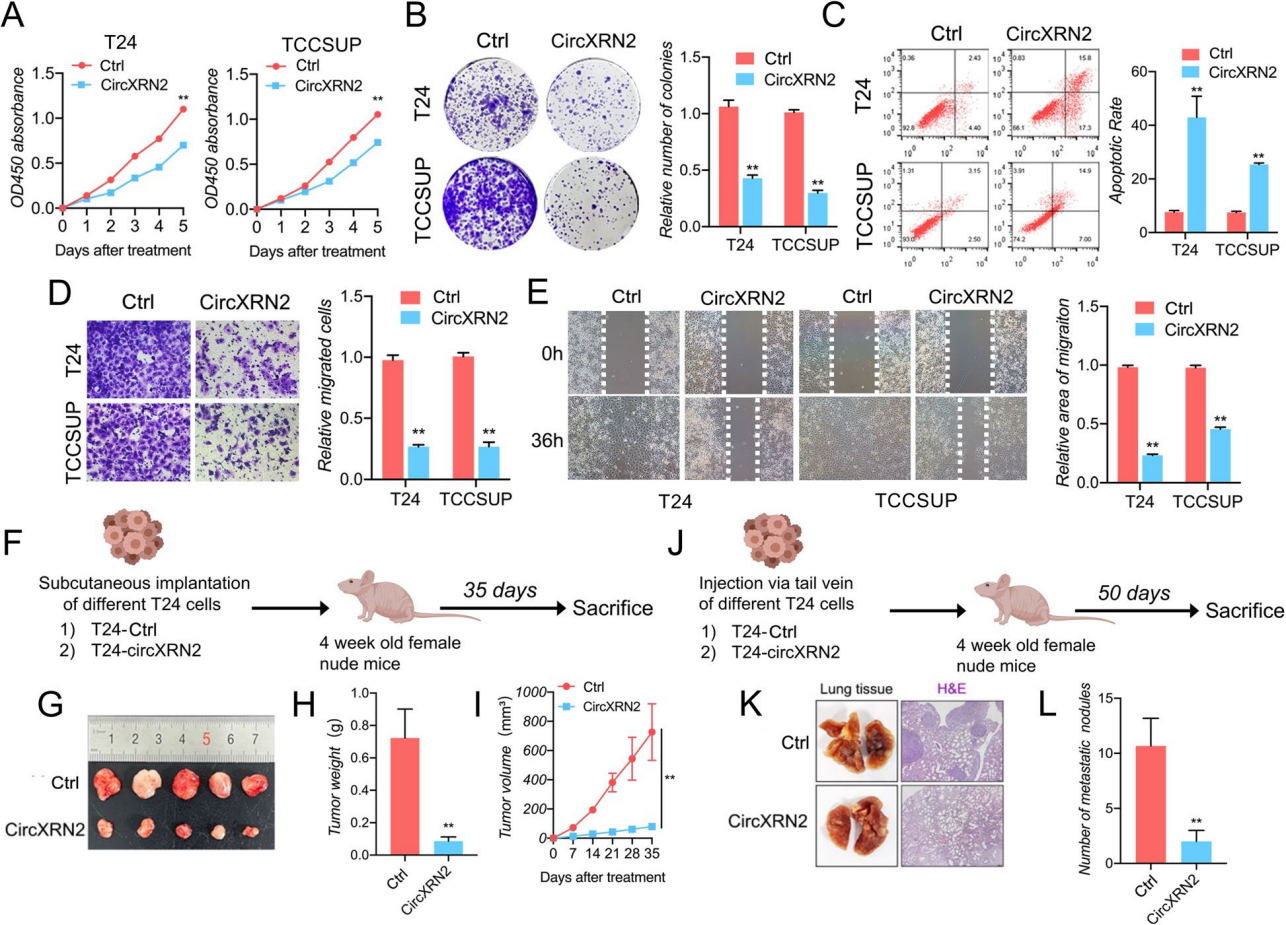
CircXRN2 inhibits the proliferation and migration of bladder cancer cells in vitro and in vivo. **a** A CCK-8 assay was conducted to evaluate cell viability in control and circXRN2-overexpressing cells.** b** CircXRN2 suppressed the colony-formation ability of bladder cancer cells.** c** CircXRN2-induced cell apoptosis in T24 and EJ cells. **d**, **e** Transwell migration assays and wound healing assays were conducted to evaluate cell migratory abilities. **f** Schematic illustration of the subcutaneous tumorigenesis model in nude mice with negative control or circXRN2-overexpressing cells. **g-i** CircXRN2 inhibited tumor growth of human bladder cancer in vivo (*n* = 5). **j** Schematic illustration of the lung metastatic model in nude mice with negative control or circXRN2-overexpressing cells.** k-l** Overexpressing circXRN2 led to fewer lung metastatic nodules than in the control group. All the data are presented as the mean ± standard deviation (*n* = 5). *P < 0.05, **P < 0.01, compared with the control group

In in vitro experiments, we performed a CCK8 assay to evaluate cell viability, and the results demonstrated the inhibitory effects of circXRN2 (Fig. 2a). Similarly, a colony formation assay was performed to test the biological function of circXRN2 in cell proliferation, which manifested a consistent trend with previous experiments (Fig. 2b). In addition, overexpression of circXRN2 also triggered cell apoptosis (Fig. 2c). In terms of cell migratory ability, as shown in Fig. 2d and e**,** circXRN2 also reduced cellular migration capacity.

In *an *in vivo study, we constructed a subcutaneous xenograft tumorigenesis model and tail vein lung metastasis model in nude mice. The results indicated that circXRN2 could reduce the growth rate and weight of subcutaneous tumors (Fig. 2f-i). Similarly, the number of metastatic nodes in the circXRN2 overexpression group was much lower than that in the negative control group (Fig. 2j-l).

Overall, our results suggested that circXRN2 suppressed the proliferation and migration of bladder cancer cells in vitro and in vivo.

### CircXRN2 prevents LATS1 from SPOP-mediated degradation

**Fig. 3:**
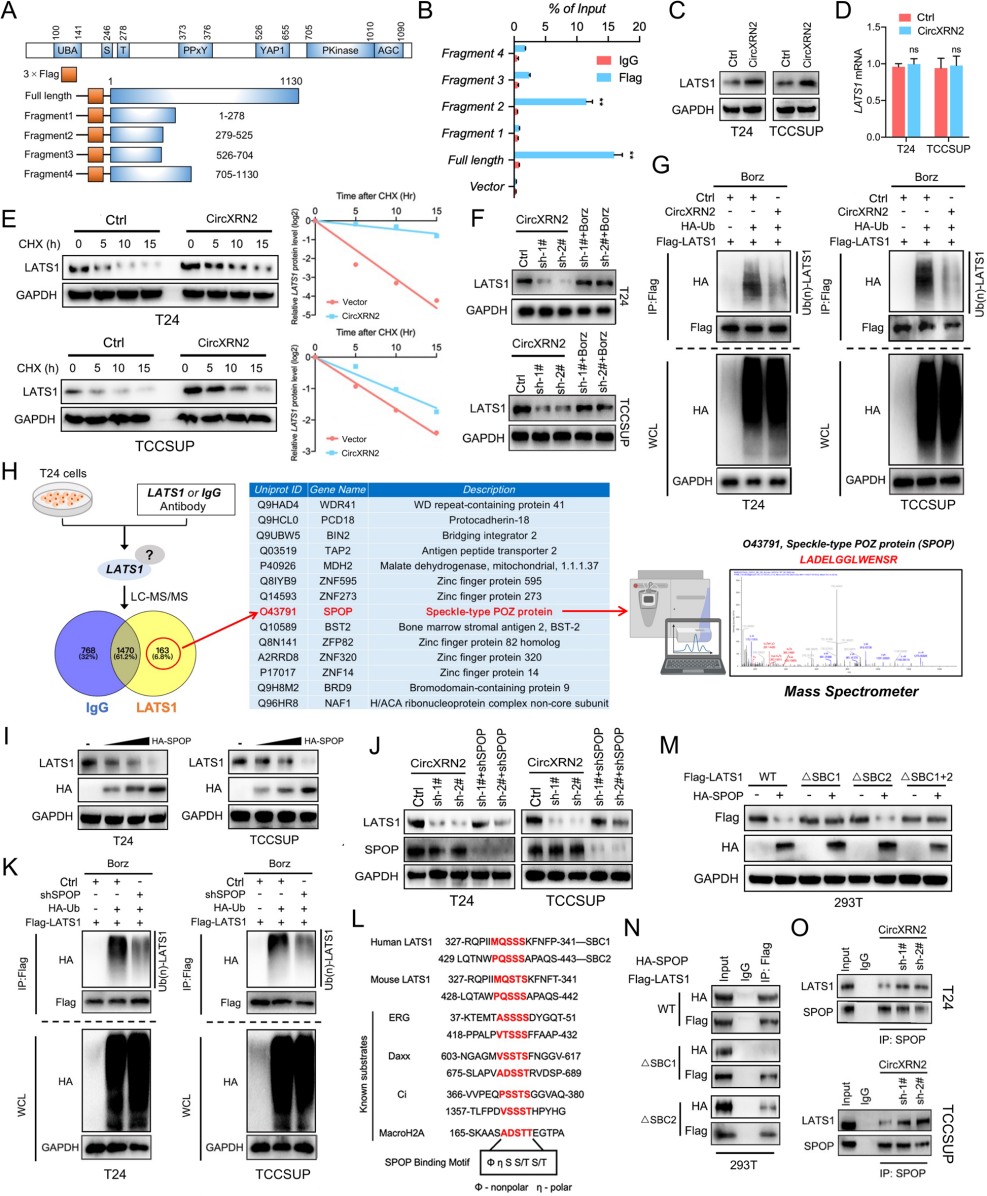
CircXRN2 prevents LATS1 from SPOP-mediated degradation by binding to its degron sequence. **a** We contrasted different fragments of the LATS1 protein to test the exact region interacting with circXRN2. A schematic illustration of the depletion map of LATS1 protein. **b** Fragment 2 and full-length LATS1 protein interacted with circXRN2, as determined by RIP assay in 293T cells. **c**, **d** CircXRN2 enhanced the expression level of LATS1 at the protein level but not the mRNA level.** e** Cycloheximide (CHX) was used to block protein synthesis. LATS1 protein levels were detected by western blotting in control and circXRN2-overexpressing bladder cancer cells. **f** Downregulation of LATS1 protein induced by circXRN2 knockdown was reversed by treatment with bortezomib (500 nM, an inhibitor of the proteasome). **g** Cells were pretreated with bortezomib (500 nM) and NEM (10 μM, an inhibitor of deubiquitinating enzyme) for 8 h. The ubiquitinated LATS1 protein levels in the control group and circXRN2-overexpressing group were detected by IP assay. **h** A mass spectrometer was used to detect the proteins interacting with LATS1. **i** LATS1 protein levels were detected by immunoblot analysis in cells transfected with incremental doses of SPOP plasmid. **j** Downregulation of LATS1 protein could be rescued by depletion of SPOP. **k** SPOP increased the ubiquitination of LATS1 protein in T24 and TCCSUP cells. **l** Schematic illustration of the sequence alignment of the LATS1 protein with the SPOP-binding degron among known substrates. **m** Flag-LATS1-containing wild-type or mutant SBCs and HA-SPOP were transfected into 293T cells. Western blotting indicated that mutation of SBC1 led to remarkable blockade of LATS1 degradation mediated by SPOP, while depletion of SBC2 had little effect. **n** Co-IP results showed that wild-type LATS1 could bind to SPOP, but the interaction of SBC1-mutant LATS1 with SPOP was almost completely diminished. Western blotting was performed to determine the expression levels of Flag-LATS1 and HA-SPOP. **o** The interactions between LATS1 and SPOP in control and circXRN2 knockdown cells were verified by Co-IP assay. All the data are presented as the mean ± standard deviation (*n* = 3). *P < 0.05, **P < 0.01

To unmask the interactions between circXRN2 and LATS1, we constructed plasmids expressing different fragments of LATS1 and transfected them into 293T cells (Fig. 3a). RIP assays showed that fragment 2 (279 aa-525 aa) of the LATS1 protein could interact with circXRN2 in 293T cells and BCa cells (Fig. 3b and Fig. S5a). Next, we found that overexpression of circXRN2 upregulated LATS1 at the protein level but not the mRNA level (Fig. 3c and d). According to the above results, we hypothesized that circXRN2 might modulate LATS1 protein levels via posttranslational modification. To confirm this conjecture, we treated negative control and circXRN2-overexpressing cells with cycloheximide (CHX, an inhibitor of eukaryotic protein synthesis), and the results showed that circXRN2 could prolong the half-life of the LATS1 protein (Fig. 3e). In addition, bortezomib (an inhibitor of the proteasome) restored the expression level of LATS1 protein in circXRN2-deficient tumor cells (Fig. 3f). Next, we performed IP assays to detect the ubiquitylation of LATS1 protein in different cells. As shown in Fig. 3g, under treatment with bortezomib and N-ethylmaleimide (NEM, an inhibitor of deubiquitinating enzymes), less LATS1 protein was ubiquitinated in circXRN2-overexpressing cells. Taken together, these results confirmed that circXRN2 modulated LATS1 protein levels in bladder cancer cells by regulating posttranscriptional ubiquitylation.

A recent study reported that LATS1 is a potential substrate of speckle-type POZ (SPOP, an E3 ubiquitin ligase adapter), and SPOP promotes ubiquitylation and degradation of LATS1 [29]. Therefore, to verify that SPOP was involved in the ubiquitylation and degradation of LATS1 in human bladder cancer cells, we first performed IP followed by mass spectrometric detection with LATS1 antibodies in T24 cells. As shown in Fig. 3h, LATS1 interacted with the SPOP protein in bladder cancer cells. Next, we transfected HA-tagged SPOP at different concentrations into bladder cancer cells. The results showed that SPOP downregulated LATS1 protein in a dose-dependent manner (Fig. 3i). Interestingly, SPOP knockdown significantly abolished the downregulation of LATS1 protein induced by circXRN2 deficiency (Fig. 3j). Moreover, an IP assay showed that SPOP knockdown markedly decreased the ubiquitylation of the LATS1 protein (Fig. 3k). In light of these results, we wondered whether SPOP participated in the degradation of LATS1 protein regulated by circXRN2.

It has been well proven that the vast majority of SPOP substrates share the SPOP-binding consensus motif F-P-S–S/T-S/T (“F” represents nonpolar, and “P” represents polar) [30]. According to previous studies, as shown in Fig. 3l, there are two putative SPOP-binding motifs or “degrons” located in the N-terminus of the LATS1 protein (SBC1: 327- MQSSS- 341; SBC2: 429- PQSSS- 443) [29]. In our previous results, circXRN2 interacted with Fragment 2 (279 aa-525 aa) of LATS1, which contained SBC1 and SBC2. Therefore, we speculated that circXRN2 and SPOP might competitively interact with the LATS1 protein to regulate its degradation. For further verification, by transfecting wild-type or mutant LATS1 plasmids into 293T or bladder cancer cells and confirmed that mutation of SBC1 led to remarkable blockade of LATS1 degradation mediated by SPOP, while depletion of SBC2 had little effect (Fig. 3m and Fig. S5b). Meanwhile, co-IP results showed that wild-type LATS1 could bind to SPOP, but the interaction of SBC1-mutant LATS1 with SPOP was almost completely diminished (Fig. 3n and Fig. S5c). Finally, as shown in Fig. 3o, we found that knockdown of circXRN2 promoted the interaction between SPOP and LATS1 in bladder cancer cells. In summary, our results demonstrated that circXRN2 prevents LATS1 from SPOP-mediated degradation.

### CircXRN2 activates the Hippo signaling pathway to regulate biological functions

In previous experiments, we confirmed that circXRN2 could bind to degrons on the LATS1 protein and inhibit the ubiquitination mediated by SPOP, thus stabilizing the endogenous LATS1 protein. As the vital upstream molecule in the Hippo pathway, LATS1 activates the signaling axis by phosphorylating TAZ/YAP [31]. Therefore, to verify that circXRN2 performs its biological functions through the Hippo pathway, we first validated that when the Hippo pathway was activated after overexpressing circXRN2, the corresponding protein levels were altered accordingly (Fig. S1a). In addition, immunofluorescence results suggested that circXRN2 increased the retention of TAZ/YAP in the cytoplasm, which also indicated the activation of the Hippo signaling pathway (Fig. S1b). Functionally, TAZ/YAP deficiency severely suppressed cell proliferation and growth rates and led to cell apoptosis at the same time (Fig. S1c-e). Transwell and wound healing assays indicated that knockdown of TAZ/YAP impaired cell migratory capacity (Fig. S1f and g). Taken together, these results support that circXRN2 activates the Hippo signaling pathway and modulates the downstream biological behaviors of bladder cancer cells.

### CircXRN2 serves as a suppressor of glycolysis and lactate production in bladder cancer

**Fig. 4:**
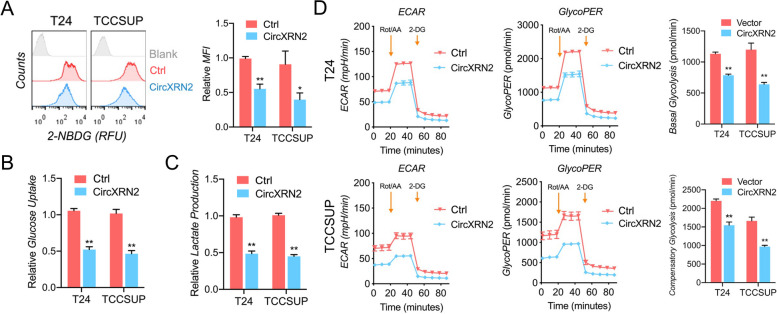
CircXRN2 is a negative regulator of glycolysis in human bladder cancer cells. **a** Control or circXRN2-overexpressing bladder cancer cells were incubated with 2-NBDG (a fluorescent D-glucose analog) and subjected to flow cytometry to monitor glucose uptake. **b** Overexpression of circXRN2 decreased the uptake of glucose in T24 and TCCSUP cells. **c** Lactate production was suppressed in the presence of circXRN2.** d** Seahorse metabolic analysis showed that circXRN2 significantly inhibited glycolysis in bladder cancer cells, which was marked by a decreased glycoPER (glycolytic proton efflux rate), basal glycolysis rate and compensatory glycolysis rate. All the data are presented as the mean ± standard deviation (*n* = 3). *P < 0.05, **P < 0.01

Many studies have reported the regulatory role of the Hippo pathway in cell glycolytic metabolism [32–34]. Given the close relationship between circXRN2, LATS1 and the Hippo pathway, we hypothesized that circXRN2 plays a key role in glycolysis in bladder cancer cells. To validate this speculation, we overexpressed circXRN2 and performed 2-NBDG uptake detection, glucose uptake assay and lactate production determination. The above experiments proved that circXRN2 reduced glucose uptake and lactate production in T24 and TCCSUP cells (Fig. 4a-c). The results of Seahorse glycolytic rate analysis indicated that glycoPER, basal glycolysis and compensatory glycolysis in bladder cancer cells overexpressing circXRN2 were lower than those in normal tumor cells (Fig. 4d). Taken together, circXRN2 plays a negative role in glycolysis and lactate metabolism in T24 and TCCSUP cells.

### The Hippo pathway modulates glycolysis and lactate production in bladder cancer cells

To further confirm that the circXRN2/Hippo axis regulates glucose metabolism, we knocked down TAZ and YAP in T24 and TCCSUP cells and used a series of experiments to evaluate glycolysis in differently treated groups. Similarly, we found a remarkable decrease in the uptake of 2-NBDG by tumor cells after knockdown of TAZ/YAP by flow cytometry (Fig. S2a). Glucose uptake assay (Fig. S2b) and lactate production detection assay (Fig. S2c) also indicated that the Hippo signaling pathway is crucial for glycolysis in bladder cancer cells. In addition, through Seahorse glycolytic rate analysis, the effects of TAZ/YAP on glycoPER, basal glycolysis and compensatory glycolysis in bladder cancer cells also showed the same trend (Fig. S2d).

### CircXRN2 activates the Hippo pathway by stabilizing LATS1 to regulate bladder cancer progression and glycolysis and lactate production

**Fig. 5:**
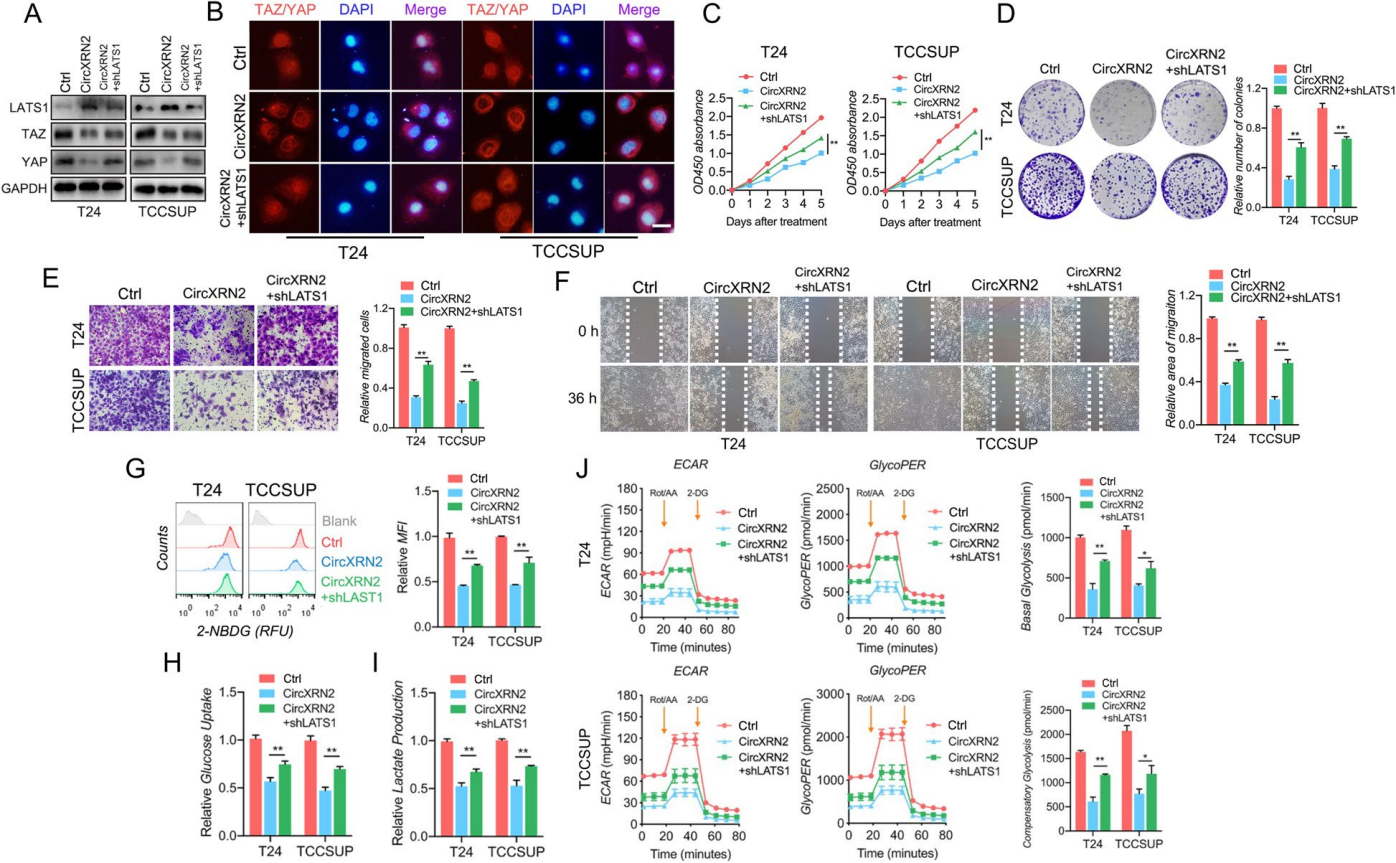
CircXRN2 activates the Hippo pathway through LATS1 to regulate cell proliferation, migration and glycolysis in human bladder cancer. **a** The expression levels of LATS1, TAZ and YAP were determined in different cells. **b** CircXRN2 altered the location of TAZ/YAP in T24 and TCCSUP cells, and knockdown of LATS1 reversed the effect of circXRN2 on TAZ/YAP. Scale bar: 50 μm. **c** CCK-8 assay showed that shLATS1 alleviated the suppression of cell viability induced by circXRN2. **d** Control, circXRN2-overexpressing and circXRN2 + shLATS1 cells were used for colony formation assays. **e, f** Cell migratory ability was impaired by circXRN2 and rescued by depletion of LATS1, as validated by Transwell migration and wound healing assays. **g-i** CircXRN2-induced inhibition of glucose uptake and lactate production was reversed by elimination of LATS1. **j** Cells were subjected to a Seahorse metabolic analyzer to determine the glycolytic rate. The glycoPER, basal glycolytic rate and compensatory glycolytic rate were higher in the circXRN2 overexpression group along with the shLATS1 group than in the circXRN2 overexpression group. All the data are presented as the mean ± standard deviation (*n* = 3). *P < 0.05, **P < 0.01

To confirm that the circXRN2/LATS1 axis regulates various biological functions and glucose metabolism in bladder tumor cells, we knocked down LATS1 protein in cells overexpressing circXRN2. Immunoblot analysis showed the protein levels of LATS1, TAZ and YAP (Fig. 5a), and the intracellular location of TAZ/YAP was confirmed by immunofluorescence (Fig. 5b). The results of the CCK-8 assay and colony formation assay showed that depletion of LATS1 reversed the inhibitory effects on cell viability and proliferation induced by circXRN2 (Fig. 5c and d). Meanwhile, cell migration was determined by Transwell assay and wound healing assay. Compared with circXRN2-overexpressing cells, those transfected with shLATS1 were more capable of migration (Fig. 5e and f).

Additionally, the glucose uptake capacity and lactate production of the control group, circXRN2-overexpressing group, and circXRN2 + shLATS1 group were determined by 2-NBDG uptake measurement, glucose uptake assay, and lactate production detection, respectively. Figure 5g-i shows that shLATS1 alleviated the suppression of glycolytic metabolism caused by circXRN2. Subsequently, glycoPER, basal glycolysis and compensatory glycolysis rates of different cells were analyzed and calculated by the Seahorse metabolic analyzer, and the results also supported the above conclusions (Fig. 5j).

### Histone lactylation modulated by circXRN2 promotes tumorigenesis of bladder cancer

**Fig. 6:**
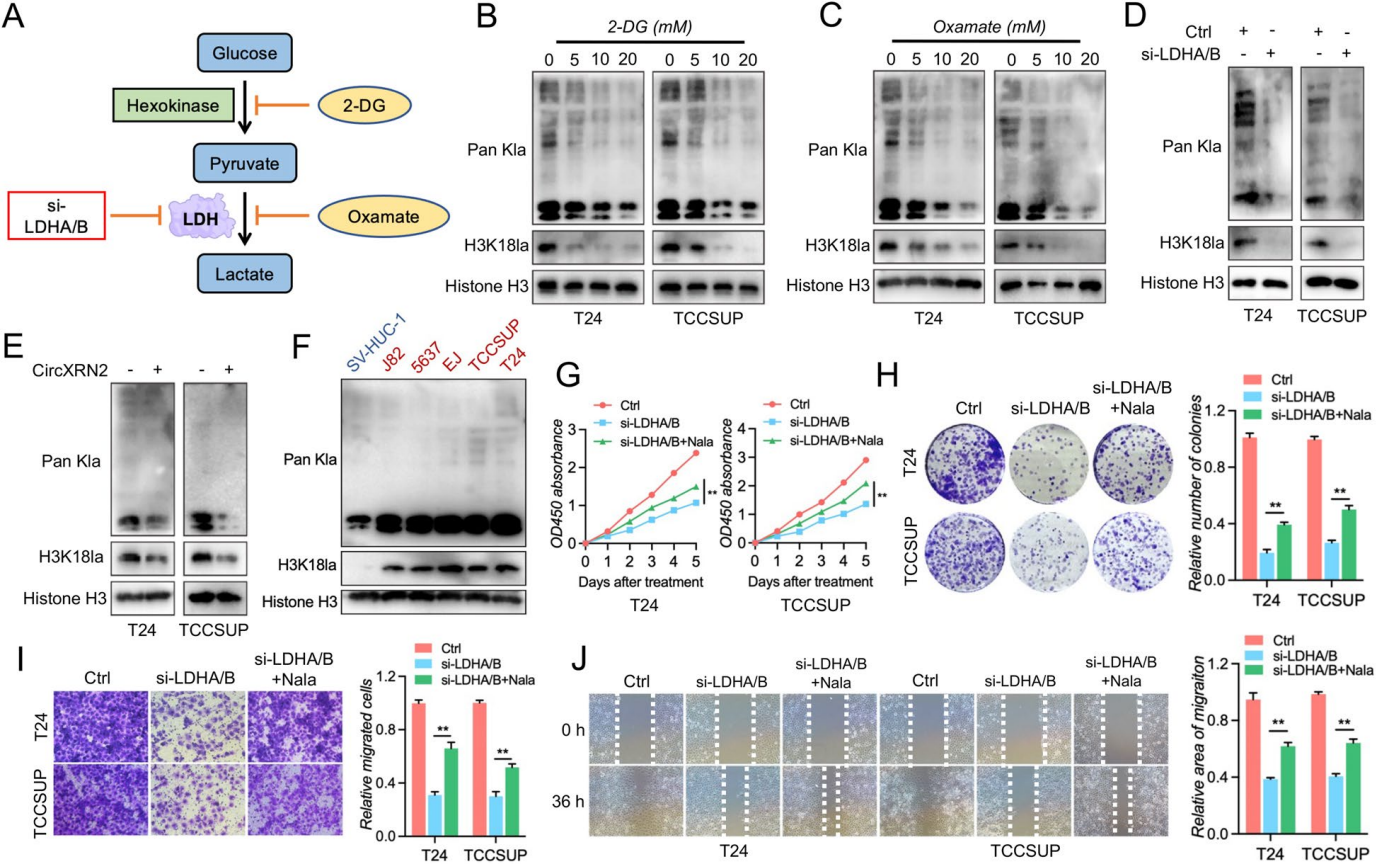
CircXRN2-mediated H3K18 lactylation promotes tumor progression in human bladder cancer. **a** A schematic illustration of glycolysis is presented, and the methods used to inhibit the production of lactate and histone lactylation in this research are indicated. **b**, **c** Cells were treated with 2-DG or oxamate (glycolysis inhibitors) at different concentrations for 24 h. Cells were collected for measuring the level of Pan Kla and H3K18 lactylation. **d** Immunoblots were carried out to determine the effect of LDH depletion on Pan Kla and H3K18 lactylation. **e** CircXRN2 suppressed intracellular Pan Kla and H3K18 lactylation in T24 and TCCSUP cells. **f** Pan Kla and H3K18 lactylation were evaluated in SV-HUC-1 and bladder cancer cell lines. **g** A CCK-8 assay was used to determine the viability of LDH-deficient bladder cancer cells. **h** Cell proliferation and colony formation ability were measured by colony formation assay. **i**, **j** The migratory ability of LDH-deficient cells was evaluated by Transwell migration assay and wound healing assay. All the data are presented as the mean ± standard deviation (*n* = 3). *P < 0.05, **P < 0.01, compared with the control group

Histone lactylation was newly discovered by Zhang et al. in 2019 and relies on intracellular lactate produced by glycolysis or other metabolic processes [26]. Therefore, we employed 2-deoxy-D-glucose (2-DG, a glycolysis inhibitor) and oxamate (a lactate dehydrogenase inhibitor) to confirm the relationship between glycolysis, global lactylation and histone lactylation in bladder cancer cells. As shown in Fig. 6b-d, the levels of global lactylation and H3K18 lactylation were decreased by 2-DG and oxamate in a dose-dependent manner and were inhibited completely by LDH depletion. The knockdown efficiency of LDHA and LDHB was verified by western blotting (Fig. S4). Considering the effects of circXRN2 on glycolysis and lactate production, we hypothesized that circXRN2 might be involved in histone lactylation in bladder cancer. As we hypothesized, the results in Fig. 6e show that circXRN2 markedly reduced global lactylation and H3K18 lactylation levels. Notably, global lactylation and H3K18 lactylation were aberrant in bladder cancer cell lines, indicating their potential roles in tumorigenesis (Fig. 6f).

Furthermore, to validate the biological functions of histone lactylation in bladder cancer, we eliminated endogenous LDHA and LDHB to reduce global lactylation and H3K18 lactylation levels and replenished sodium lactate for rescue experiments. CCK-8 and colony formation assays indicated that histone lactylation played a vital role in cell proliferation and colony formation ability (Fig. 6g and h). In addition, simultaneous elimination of LDHA and LDHB also impaired cell migratory ability in T24 and TCCSUP cells (Fig. 6i and j). Moreover, sodium lactate attenuated the suppression of tumor cell proliferation (Fig. 6g and h) and migration (Fig. 6i and j) induced by LDHA/B deficiency. Taken together, these results indicated that histone H3K18 lactylation was modulated by circXRN2 and was involved in the tumorigenesis of bladder cancer.

### LCN2 is a target of H3K18 lactylation and acts as an oncogene in bladder cancer

**Fig. 7:**
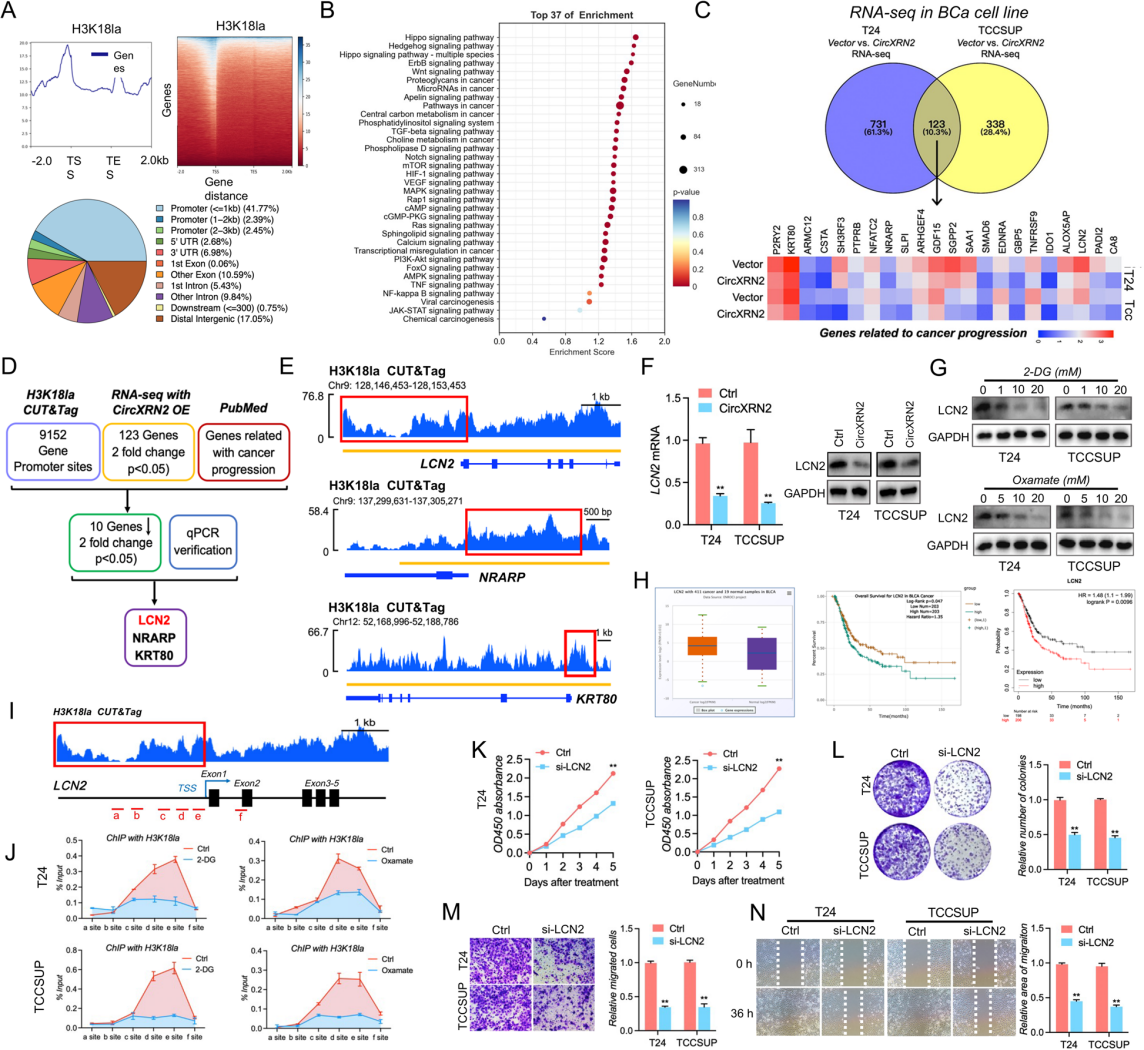
LCN2 is a target of H3K18 lactylation and acts as an oncogene. **a** CUT&Tag was performed with H3K18la antibodies in T24 cells. H3K18la could be enriched in the promoter region of numerous genes. **b** KEGG database analysis was carried out in H3K18la-related genes. **c** Transcriptome sequencing was performed in control and circXRN2-overexpressing T24 and TCCSUP cells. **d** Flow diagram confirming the downstream target of H3K18la. **e** H3K18la peaks in the promoter regions of LCN2, NRARP and KRT80. **f** LCN2 mRNA and protein levels were measured in circXRN2-overexpressing cells. **g** Different concentrations of 2-DG and oxamate were added to T24 and TCCSUP cells, and LCN2 protein levels were measured by immunoblotting. **h** LCN2 mRNA was upregulated in bladder cancer and was closely related to the overall survival time of bladder cancer patients in the TCGA database and K‒M plotter. **i** Primers targeting different fragments of the LCN2 gene are indicated, and the c, d, and e sites are H3K18la peaks of the LCN2 promoter region in CUT&Tag. **j** ChIP assay following qPCR was used to detect the binding status of H3K18la in the LCN2 promoter region in bladder cancer cells treated with or without glycolysis inhibitors. **k-n** The roles of LCN2 in cell proliferation and migration were verified by CCK-8 assay, colony formation assay, Transwell migration assay and wound healing assay. All the data are presented as the mean ± standard deviation (*n* = 3). *P < 0.05, **P < 0.01, compared with the control group

To unmask the regulatory mechanism of H3K18 lactylation, CUT&Tag was performed with ChIP-grade H3K18la antibody and IgG in T24 cells (Supplementary files 6 and 7). As shown in Fig. 7a, H3K18la could be enriched in the promoter region of numerous genes. Meanwhile, KEGG database analysis showed that H3K18la-related genes in our CUT&Tag sequencing were enriched in multiple signaling pathways regulating tumorigenesis (Fig. 7b), indicating the pivotal role of H3K18la in human bladder cancer progression. We then performed transcriptomic sequencing in T24 and TCCSUP cells with or without overexpression of circXRN2 and combined these results with CUT&Tag data and the PubMed database, and LCN2, NRARP and KRT80 were selected as candidate genes (Fig. 7c and d, Supplementary files 4 and 5). The signals of enriched H3K18la or IgG in the promoter regions of LCN2, NRARP and KRT80 can be seen in Fig. 7e and Fig. S6. We next validated the expressions of the above targets at mRNA level in circXRN2-overexpressing cells, and the results showed that LCN2 could be remarkably regulated by circXRN2 (Fig. S7a). Then, the expression levels of LCN2 mRNA and LCN2 protein were verified in T24 and TCCSUP cells overexpressing circXRN2. The results showed LCN2 was significantly downregulated by circXRN2 (Fig. 7f). Moreover, glycolysis inhibitors also modulated the expression of LCN2 at the protein level (Fig. 7g). Recently, LCN2 was identified as a promotor of cancer progression [35–37]. In addition, we found that LCN2 was upregulated in bladder cancer tissues compared to normal tissues and was closely related to bladder cancer progression in the TCGA database and Kaplan‒Meier Plotter database (Fig. 7h). Based on the above evidence, LCN2 was selected for the following research. According to our previous CUT&Tag sequencing, specific primers targeting different regions of the LCN2 promoter were designed, and ChIP assays showed that H3K18la was enriched in the LCN2 promoter. Notably, the interaction between H3K18la and the LCN2 promoter could be reduced by glycolysis inhibitors and circXRN2, further indicating the key role of H3K18la in LCN2 transcription (Fig. 7i, j and Fig. S7b). Finally, we explored the biological functions of LCN2 in LCN2-deficient T24 and TCCSUP cells. As shown in Fig. 7k-n, depletion of LCN2 dramatically attenuated the proliferation, colony formation and migration of tumor cells. In summary, our results suggested that LCN2 is directly regulated by H3K18 lactylation and serves as an oncogene in human bladder cancer.

### H3K18 lactylation promotes bladder cancer progression through LCN2 expression

Since H3K18la could regulate LCN2 expression, rescue experiments were carried out to verify the interaction between H3K18la and LCN2 in bladder cancer progression. Specifically, we reduced the level of histone lactylation by eliminating LDHA and LDHB and then replenished LCN2 in LDH-deficient cells. As expected, LCN2 could partially counteract the antitumor effects on cell viability (Fig. S3a), colony formation (Fig. S3b) and migration (Fig. S3c and d) mediated by si-LDHA/B. In addition, overexpression of LCN2 also promoted cell proliferation, colony formation and migration in T24 and TCCSUP cells, confirming the oncogenic effects of LCN2 (Fig. S3a-d).

### The circXRN2-LATS1 axis inhibits tumor growth and metastasis of bladder cancer cells in vivo

**Fig. 8:**
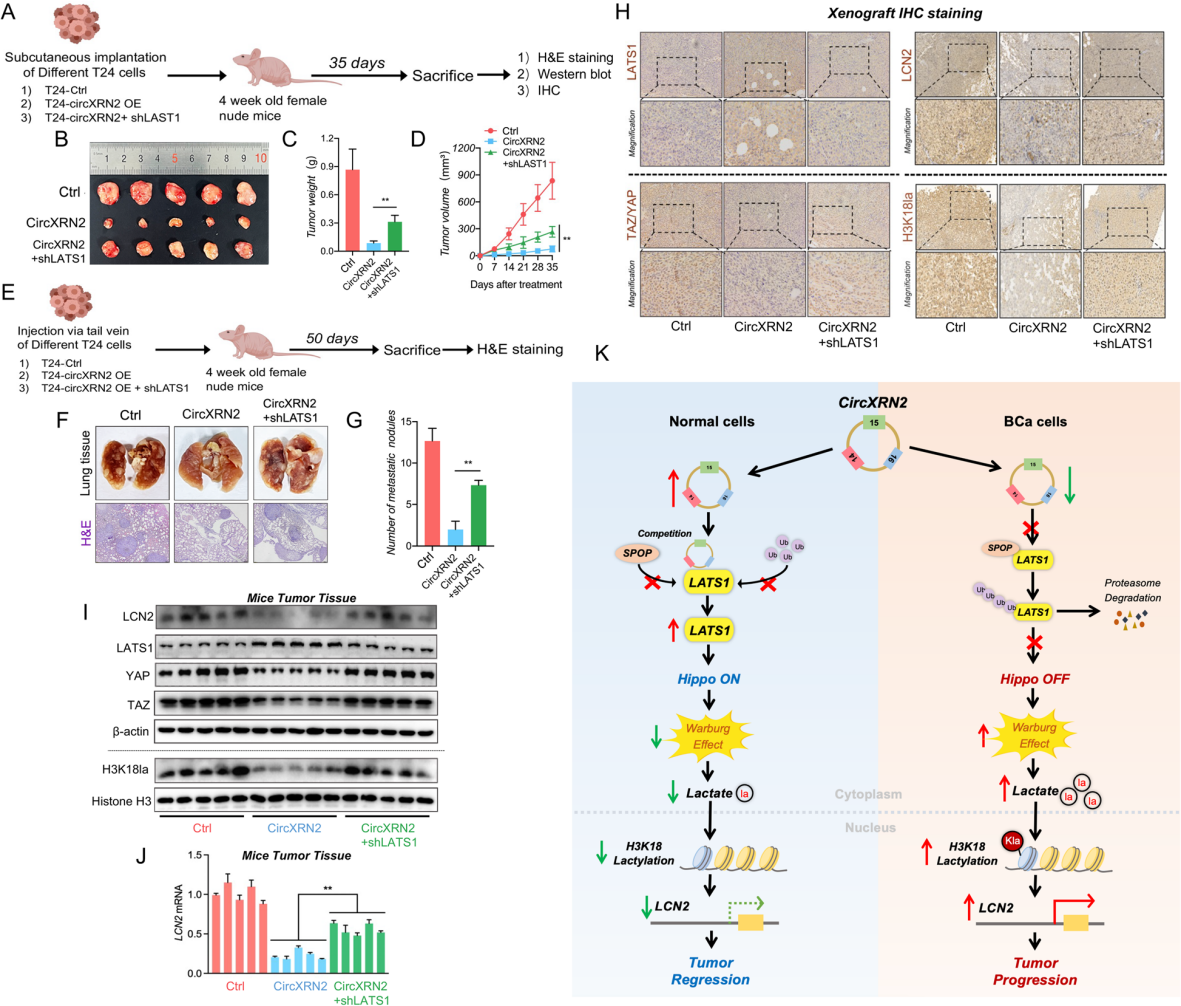
CircXRN2/LATS1 axis inhibits tumor growth and metastasis of bladder cancer in vivo. **a** T24 cells (6 × 10^6^) transfected with control, circXRN2 or circXRN2 and shLATS1 were injected subcutaneously to establish a tumorigenesis model in nude mice. **b**, **c** At Day 35 after treatment, all mice were sacrificed, and tumors were dissected and photographed. Tumor weight was also recorded. **d** The parameters of subcutaneous tumors were measured and recorded every 7 days. We calculated the volume of the tumor according to the formula below: tumor volume = 0.5 × length × width × width (mm^3^).** e** We also established a lung metastasis model by injecting 5 × 10^6^ different T24 cells into the tail vein to evaluate the ability of cells to metastasize in vivo. **f**, **g** Fifty days later, mice were sacrificed, and lung tissues were photographed and subjected to H&E staining. The number of lung metastatic nodules was counted. **h** Immunohistochemistry was used to visualize and compare the protein levels of LATS1, TAZ/YAP, LCN2 and H3K18la in tumors collected from the control, circXRN2 overexpression and circXRN2 with shLATS1 groups. Scale bar: 20 μm. **i** Immunoblotting was used to determine the protein levels of LATS1, TAZ/YAP, LCN2 and H3K18la in tumors collected from the control, circXRN2 overexpression and circXRN2 with shLATS1 groups. **j** The expression levels of LCN2 mRNA were measured by qRT‒PCR.** k** Schematic illustration of the current study: circXRN2 suppresses tumor progression driven by H3K18 lactylation in human bladder cancer by activating the Hippo signaling pathway. All the data are presented as the mean ± standard deviation (*n* = 5 in the tumorigenesis model, *n* = 3 in the metastatic model). *P < 0.05, **P < 0.01, compared with the circXRN2 overexpression group

To verify whether the circXRN2-LATS1 axis inhibits tumor growth and metastasis of bladder cancer cells in vivo, we subcutaneously injected cells with different treatments (control, circXRN2 overexpression, circXRN2 + shLATS1) into nude mice. Similar to in vitro cell experiments, overexpression of circXRN2 significantly reduced the tumor growth rate, and knockdown of LATS1 significantly attenuated the inhibitory effect caused by circXRN2 (Fig. 8a-d). We also established a nude mouse lung metastasis model by injecting different T24 cells into the tail vein to evaluate the ability of cells to metastasize in vivo. As demonstrated in Fig. 8e-g, overexpression of circXRN2 significantly reduced lung metastatic nodules compared with the normal control group, while the tumor nodules were significantly increased in cells treated with circXRN2 overexpression along with knockdown of LATS1. In addition, we detected the expression levels of LATS1, TAZ/YAP, LCN2 and H3K18la in tumors of different treatment groups by immunohistochemistry and western blotting, and the results were consistent with those of previous experiments (Fig. 8h-i). Meanwhile, the expression levels of LCN2 mRNA were evaluated in different tumors (Fig. 8j). In conclusion, our findings illuminated that circXRN2 suppresses tumor progression mediated by H3K18 lactylation in human bladder cancer by activating the Hippo signaling pathway.

## Discussion

In general, due to the huge amount of information in the genome and variable coding mechanisms, the third nucleotide of the codons in the exons often varies. However, the exons involved in the formation of the relevant circRNA are on the contrary, and their sequences are evolutionarily conserved, which suggests that circRNA plays an important noncoding role in eukaryotic cells [38]. Existing studies have only clarified and excavated a small part of the biological functions of circRNAs, in which most studies have reported that they act as microRNA sponges or decoys [12, 13]. For example, our previous studies demonstrated that circRIMS1 and circPPP1CB could serve as miRNA sponges to regulate bladder cancer progression [18, 19]; circRHOBTB3 is an endogenous competitive RNA for miR-654-3p to suppress gastric cancer growth [39]; the circNSD2/miR-199b-5p regulatory axis was confirmed to be involved in the progression of colorectal cancer [40] and hsa_circ_0003258 regulates prostate cancer progression by binding to miR-653-5p and modulating IGF2BP3 expression [41]. Nevertheless, current studies rarely investigate novel molecular mechanisms and regulatory models of circRNAs in human bladder cancer, and further studies are urgently needed.

First, our study demonstrated that circXRN2 could interact with the LATS1 protein and was dysregulated in bladder cancer cell lines and surgical samples. Meanwhile, circXRN2 suppressed cell proliferation and migration in vitro and in vivo. In addition, we validated that circXRN2 overexpression upregulated LATS1 protein levels by inhibiting its ubiquitylation and degradation.

Apart from functioning as endogenous competitive RNAs, circRNAs can also serve as scaffolding molecules that modulate protein‒protein interactions and the stability of proteins and then regulate various signaling pathways and biological processes. For example, by forming the circFOXO3/p21/CDK2 ternary complex, circFOXO3 enhances the interaction between p21 and CDK2 and reduces CDK2 phosphorylation activity [17]. A similar effect is also found in circNFIX, which strengthens the interplay between Y-box binding protein 1 (YBX1) and NEDD4-like E3 ubiquitin ligase (Nedd4l), leading to YBX1 ubiquitination [42]. A recent study showed that SPOP increases ubiquitination of the LATS1 protein and promotes its degradation in kidney cancer [29]. SPOP, as a subunit of the Cullin-RING E3 ligase, exerts its biological functions by recognizing specific substrates for ubiquitination [43]. In light of this evidence, we further demonstrated that circXRN2 interacts with the SPOP-binding degron on the LATS1 protein and prevents LATS1 from undergoing SPOP-mediated ubiquitination and degradation.

The Hippo signaling pathway consists of a kinase cascade and two executors (TAZ and YAP) [31]. As research has progressed, increasing attention has been garnered on the regulation of tumorigenesis by mediating the Hippo pathway. For instance, the E3 ubiquitin ligase PARK2 promoted esophageal squamous carcinoma progression by inhibiting the Hippo/YAP signaling pathway [44]. MYBL2 impairs the Hippo signaling pathway and regulates castration resistance and metastasis in prostate cancer [45]. In addition, the Hippo pathway also participates in tumorigenesis in multiple human cancers [46–48]. Considering that LATS1 is a key molecule in the kinase cascade of the Hippo pathway, we further demonstrated that circXRN2 activates the Hippo signaling pathway, thereby regulating biological functions in bladder cancer cells.

Histones are a type of protein that binds to DNA in eukaryotic nucleosomes and regulates DNA-templated processes. Usually, there are a large number of posttranslational modifications (PTMs) at the N-terminal tail, such as methylation, acetylation, and succinylation [49]. In 2019, histone lactylation was discovered and reported for the first time by Zhang et al. [26]. Histone lactylation is derived from lactate produced by cellular metabolism and modulates biological processes by promoting gene transcription. Different from normal cells, glycolysis, instead of aerobic oxidation, is preferred for tumor cells to acquire enough energy for maintaining their rapid growth and survival, even under well-oxygenated conditions. The above biological phenomenon is also termed the Warburg effect and is one of the metabolic characteristics in tumor cells [50]. Therefore, tumor cells will produce and accumulate more lactate than normal cells, suggesting that histone lactylation in tumors is more likely to be aberrant and worthy of investigation. To date, emerging literature has shown that histone lactylation drives tumor progression in various types of human cancer. Numb/Parkin-mediated mitochondrial fitness governs the differentiation of prostate cancer and lung adenocarcinoma cells via regulation of histone lactylation [51]. Meanwhile, H3K18la promotes METTL3-mediated m^6^A modification to enhance the immunosuppressive effect of tumor-infiltrating myeloid cells [52]. H3K18la can also modulate the expression of YTHDF2 to participate in the progression of ocular melanoma [53]. Nevertheless, the exact roles of histone lactylation in human bladder cancer remain to be discovered. In the current research, we discovered the vital role and aberrant expression level of H3K18 lactylation in human bladder cancer tumorigenesis. Until now, it has been well known that histone lactylation and acetylation at lysine residues engage in a competitive relationship and serve as indicators for the levels of lactate and acetyl-CoA [54]. The fate of cells, whether they lean toward malignancy or not, hinges on the outlet of pyruvate committed to lactate or acetyl-CoA generation as the end product of glycolysis. While increased acetyl-CoA synthesis propels the tricarboxylic acid (TCA) cycle toward the efficient utilization of glucose and the generation of ATP, tumor cells are distinguished by their heightened production of lactate, which fuels uncontrolled cell growth by facilitating excessive biomass production [55]. Moreover, histone lactylation exhibits distinct temporal dynamics and exerts different effects on gene transcription when compared to histone acetylation. However, the investigation of histone lactylation, its interaction with histone acetylation, and other posttranslational modification events is still in its early stages. In forthcoming research, our focus will be on delving deeper into the underlying mechanisms governing these histone epigenetic marks and their phenotypic manifestations, thus broadening our comprehension of the treatment of human bladder cancer.

Notably, dysregulation of the Hippo pathway induces metabolic reprogramming in human cancers, including glucose metabolism, glutamine metabolism, fatty acid metabolism and other metabolites [56]. A study reported that METTL3 regulates glycolysis by regulating m6A methylation of the key molecule of the Hippo pathway, LATS1, in breast cancer [57]. Moreover, the HIF-1α/YAP signaling axis modulates glucose/iodine metabolism in papillary thyroid cancer progression [58]. Xu and colleagues verified that LINC00941 enhances the Warburg effect of pancreatic cancer cells by modulating the Hippo pathway [59]. In light of this evidence, our research also confirmed for the first time that circXRN2 suppressed H3K18 lactylation by activating the Hippo pathway and that H3K18 lactylation exerted its oncogenic functions by enhancing oncogene LCN2 expression. However, the underlying mechanism by which LCN2 promotes bladder cancer needs to be explored in the future.

## Conclusion

This current study explores a novel molecular mechanism of circRNA and provides concrete evidence for circRNA and histone lactylation modulating tumor progression in human bladder cancer. Specifically, we confirmed that circXRN2 suppresses bladder cancer progression by regulating H3K18 lactylation. Mechanistically, circXRN2 binds to the LAST1 protein to protect it from SPOP-mediated ubiquitylation and degradation, which subsequently activates the Hippo signaling pathway to inhibit H3K18 lactylation. Our findings will contribute to discovering new molecular targets and therapeutic strategies in human bladder cancer.

### Supplementary Information

**Additional file 1: Supplementary File 1.** The information of BCa patients.

**Additional file 2: Supplementary File 2.** circRNA_enrich by LATS1.

**Additional file 3: Supplementary File 3.** The sequences of primers and RNAs.

**Additional file 4: Supplementary File 4.** T24 circXRN2-vs-NC-RNA-seq.

**Additional file 5: Supplementary File 5.** TCCSUP circXRN2-vs-NC-RNA-seq.

**Additional file 6: Supplementary File 6.** CUT&Tag with H3K18la in T24.peak.annotation.

**Additional file 7: Supplementary File 7.** CUT&Tag with IgG in T24.peak.annotation.

**Additional file 8: Figure S1.** CircXRN2 activates the Hippo signaling pathway to suppress tumorigenesis. a. CircXRN2 increased the expression level of LATS1 and caused corresponding alterations in TAZ and YAP. b. Overexpression of circXRN2 led to cytoplasmic retention of TAZ/YAP. Scale bar: 50 μm. c. CCK-8 assay proved that the Hippo pathway was vital for cell viability. d. Deficiency of TAZ/YAP inhibited colony formation in T24 and EJ cells. e. The cell apoptosis rate was investigated by flow cytometry with Annexin V-FITC and PI staining. f. The TAZ/YAP-deficient group had fewer migrated cells than the control group in the Transwell migration assay. g. Wound healing assay showed that TAZ/YAP promoted cell migration. All the data are presented as the mean ± standard deviation (*n*=3). *P <0.05, **P<0.01, compared with the control group.

**Additional file 9: Figure S2.** The Hippo pathway modulates glycolysis in human bladder cancer. a. Glucose uptake was detected by flow cytometry in cells incubated with 2-NBDG. b. Depletion of TAZ/YAP abolished glucose uptake in T24 and TCCSUP cells. c. Impairment of the Hippo pathway reduced lactate production. d. The glycolytic rate in TAZ/YAP knockdown tumor cells was remarkably lower than that in normal tumor cells, as determined by a Seahorse metabolic analyzer. All the data are presented as the mean ± standard deviation (*n*=3). *P <0.05, **P<0.01.

**Additional file 10: Figure S3.** LCN2 attenuates antitumor effects induced by glycolysis inhibition. a. Cell viability was measured in LDH-deficient cells with or without overexpression of LCN2. b. Colony formation assay indicated the rescue effect of LCN2 on LDH silencing. c-d. Transwell migration assays and wound healing assays were performed in LDH-deficient cells with or without overexpression of LCN2. All the data are presented as the mean ± standard deviation (*n*=3). *P <0.05, **P<0.01, compared with the control group.

**Additional file 11: Figure S4.** The knockdown efficiency of LDHA/B in cells after transfection with si-LDHA/B. The expression levels of LDHA and LDHB were measured in LDH-deficient cells by western blotting to confirm the knockdown efficiency of LDHA and LDHB.

**Additional file 12: Figure S5.** The interaction between SPOP and LATS1 was verified in BCa cells. a. We contrasted different fragments of the LATS1 protein to test the exact region interacting with circXRN2 in T24 and TCCSUP cells. b. Flag-LATS1-containing wild-type or mutant SBCs and HA-SPOP were transfected into BCa cells. Western blotting indicated that mutation of SBC1 led to remarkable blockade of LATS1 degradation mediated by SPOP, while depletion of SBC2 had little effect. c. Co-IP results showed that wild-type LATS1 could bind to SPOP, but the interaction of SBC1-mutant LATS1 with SPOP was almost completely diminished. Western blotting was performed to determine the expression levels of Flag-LATS1 and HA-SPOP.

**Additional file 13: Figure S6.** The results of CUT&Tag with IgG in T24 cells. a. The enrichment of DNA fragments in CUT&Tag with H3K18la or IgG. b-d. The enrichment peaks in the promoter regions of the LCN2, NRARP and KRT80 genes are indicated.

**Additional file 14: Figure S7.** Validation of candidate genes regulated by circXRN2. LCN2, NRARP and KRT80 mRNA levels were measured in circXRN2-overexpressing cells.

**Additional file 15: Figure S8.** The results of ChIP-qPCR with H3K18 in circXRN2-overexpressing cells. ChIP assay following qPCR was used to detect the binding status of H3K18la in the LCN2 promoter region in circXRN2-overexpressing cells.

## Data Availability

The datasets used and/or analyzed in this article were included within the article and the additional files. Please contact the corresponding author for data requests.

## Abbreviations

BCa: Bladder cancer
CircRNA: Circular RNA
ShRNA: Small hairpin RNA
LATS1: Large tumor suppressor kinase 1
SPOP: Speckle-type POZ
LCN2: Lipocalin-2
IP: Immunoprecipitation
CUT&Tag: Cleavage under targets and Tagmentation
ChIP: Chromatin immunoprecipitation
FISH: Fluorescence in situ hybridization
