# Supplementary material for: CircXRN2 suppresses tumor progression driven by histone lactylation through activating the Hippo pathway in human bladder cancer
Source: Mol Cancer. 2023 Sep 8;22:151. doi: 10.1186/s12943-023-01856-1 (PMC10486081; doi:10.1186/s12943-023-01856-1)
Supplement: Supplementary file 1 — Additional file 1: Supplementary File 1. The information of BCa patients. [file 12943_2023_1856_MOESM1_ESM.docx]

**CircXRN2 suppresses tumor progression driven by histone lactylation through activating the Hippo pathway in human bladder cancer**

Bo Xie^1^, Juntao Lin^1^, Xianwu Chen^1^, Xuejian Zhou^1^, Yan Zhang^1^, Mengjing Fan^2^, Jiayong Xiang^1^, Ning He^1^, Zhenghui Hu^1^, *Feifan Wang^1^

^1^Department of Urology, The First Affiliated Hospital, Zhejiang University School of Medicine, Hangzhou, Zhejiang, 310003, R.P. China.

^2^ Department of Pathology, Sir Run Run Shaw Hospital, Zhejiang University School of Medicine, Hangzhou, Zhejiang, 310016, R.P. China.

**Correspondence:**

Feifan Wang, Department of Urology, The First Affiliated Hospital, Zhejiang University School of Medicine, Hangzhou, Zhejiang, 310003, R.P. China;

1. mail: 1322083@zju.edu.cn

**Table S1. Detailed information of our own 40 bladder cancer patients is listed**

| Patient number | Age at surgery | Gender | Grade | T | N | M | AJCC clinical stage |
| --- | --- | --- | --- | --- | --- | --- | --- |
| 1 | 69 | Female | High | Ta | N0 | M0 | Ta |
| 2 | 57 | Male | High | T2a | N0 | M0 | 2 |
| 3 | 63 | Male | High | T2 | N0 | M0 | 2 |
| 4 | 56 | Male | High | T1 | N0 | M0 | 1 |
| 5 | 75 | Female | High | T3a | N0 | M0 | 3 |
| 6 | 52 | Male | Low | T4b | N0 | M0 | 4 |
| 7 | 71 | Male | High | T1 | N1 | M0 | 4 |
| 8 | 87 | Male | High | T1 | N0 | M0 | 1 |
| 9 | 77 | Male | High | T3 | N0 | M0 | 3 |
| 10 | 65 | Male | High | T1 | N0 | M0 | 1 |
| 11 | 50 | Male | High | Ta | N0 | M0 | Ta |
| 12 | 88 | Male | High | T1 | N1 | M0 | 4 |
| 13 | 82 | Male | High | T4a | N1 | M0 | 4 |
| 14 | 63 | Male | High | T1 | N0 | M0 | 1 |
| 15 | 72 | Female | High | Ta | N0 | M0 | Ta |
| 16 | 64 | Male | High | T1 | N0 | M0 | 1 |
| 17 | 64 | Male | Low | Ta | N0 | M0 | Ta |
| 18 | 40 | Male | Low | Ta | N0 | M0 | Ta |
| 19 | 67 | Male | High | T3 | N0 | M0 | 3 |
| 20 | 72 | Male | High | T4b | N2 | M0 | 4 |
| 21 | 72 | Male | Low | Ta | N0 | M0 | Ta |
| 22 | 81 | Male | Low | Ta | N0 | M0 | Ta |
| 23 | 76 | Female | High | Ta | N0 | M0 | Ta |
| 24 | 73 | Male | Low | Ta | N0 | M0 | Ta |
| 25 | 46 | Male | Low | Ta | N0 | M0 | Ta |
| 26 | 82 | Male | High | T1 | N0 | M0 | 1 |
| 27 | 79 | Male | High | Ta | N0 | M0 | Ta |
| 28 | 33 | Male | High | T1 | N0 | M0 | 1 |
| 29 | 87 | Male | High | T1 | N0 | M0 | 1 |
| 30 | 75 | Male | Low | Ta | N0 | M0 | Ta |
| 31 | 64 | Female | Low | Ta | N0 | M0 | Ta |
| 32 | 54 | Male | Low | Ta | N0 | M0 | Ta |
| 33 | 76 | Male | Low | Ta | N0 | M0 | Ta |
| 34 | 57 | Male | High | T1 | N0 | M0 | 1 |
| 35 | 54 | Male | Low | Ta | N0 | M0 | Ta |
| 36 | 68 | Male | High | Ta | N0 | M0 | Ta |
| 37 | 40 | Male | High | T2 | N0 | M0 | 2 |
| 38 | 88 | Male | High | Ta | N0 | M0 | Ta |
| 39 | 79 | Male | Low | Ta | N0 | M0 | Ta |
| 40 | 73 | Male | High | T1 | N0 | M0 | 1 |

**Table S2.** **Detailed information of 30 bladder cancer cases of tissue microarray**

| Patient number | Age at surgery | Gender | Grade | T | N | M | AJCC clinical stage |
| --- | --- | --- | --- | --- | --- | --- | --- |
| 1 | 63 | Male | Low | Ta | N0 | M0 | Ta |
| 2 | 79 | Male | High | Ta | N0 | M0 | Ta |
| 3 | 75 | Male | Low | Ta | N0 | M0 | Ta |
| 4 | 69 | Male | High | Ta | N0 | M0 | Ta |
| 5 | 48 | Female | High | T1 | N0 | M0 | 1 |
| 6 | 70 | Male | High | T1 | N0 | M0 | 1 |
| 7 | 54 | Male | High | T1 | N0 | M0 | 1 |
| 8 | 74 | Male | High | T1 | N0 | M0 | 1 |
| 9 | 72 | Male | High | T2 | N0 | M0 | 2 |
| 10 | 61 | Female | High | T2 | N0 | M0 | 2 |
| 11 | 77 | Male | High | T2 | N0 | M0 | 2 |
| 12 | 61 | Female | High | T3 | N0 | M0 | 3 |
| 13 | 51 | Male | Low | T3 | N0 | M0 | 3 |
| 14 | 65 | Female | High | T3 | N0 | M0 | 3 |
| 15 | 61 | Female | High | T3 | N0 | M0 | 3 |
| 16 | 46 | Female | High | T3 | N0 | M0 | 3 |
| 17 | 63 | Male | High | T3 | N0 | M0 | 3 |
| 18 | 65 | Male | High | T3 | N0 | M0 | 3 |
| 19 | 69 | Male | High | T3 | N0 | M0 | 3 |
| 20 | 79 | Male | High | T3 | N0 | M0 | 3 |
| 21 | 80 | Male | High | T3 | N0 | M0 | 3 |
| 22 | 62 | Female | High | T3 | N0 | M0 | 3 |
| 23 | 60 | Male | High | T3 | N0 | M0 | 3 |
| 24 | 73 | Male | High | T3 | N0 | M0 | 3 |
| 25 | 59 | Male | High | T3 | N0 | M0 | 3 |
| 26 | 73 | Male | High | T3 | N0 | M0 | 3 |
| 27 | 61 | Male | High | T4a | N0 | M0 | 3 |
| 28 | 64 | Female | High | T3 | N1 | M0 | 4 |
| 29 | 63 | Female | Low | T3 | N1 | M0 | 4 |
| 30 | 70 | Male | High | T2 | N2 | M0 | 4 |
