# Supplementary material for: CircXRN2 suppresses tumor progression driven by histone lactylation through activating the Hippo pathway in human bladder cancer
Source: Mol Cancer. 2023 Sep 8;22:151. doi: 10.1186/s12943-023-01856-1 (PMC10486081; doi:10.1186/s12943-023-01856-1)
Supplement: Supplementary file 3 — Additional file 3: Supplementary File 3. The sequences of primers and RNAs. [file 12943_2023_1856_MOESM3_ESM.docx]

**CircXRN2 suppresses tumor progression driven by histone lactylation through activating the Hippo pathway in human bladder cancer**

Bo Xie^1^, Juntao Lin^1^, Xianwu Chen^1^, Xuejian Zhou^1^, Yan Zhang^1^, Mengjing Fan^2^, Jiayong Xiang^1^, Ning He^1^, Zhenghui Hu^1^, *Feifan Wang^1^

^1^Department of Urology, The First Affiliated Hospital, Zhejiang University School of Medicine, Hangzhou, Zhejiang, 310003, R.P. China.

^2^ Department of Pathology, Sir Run Run Shaw Hospital, Zhejiang University School of Medicine, Hangzhou, Zhejiang, 310016, R.P. China.

**Correspondence:**

Feifan Wang, Department of Urology, The First Affiliated Hospital, Zhejiang University School of Medicine, Hangzhou, Zhejiang, 310003, R.P. China;

E-mail: 1322083@zju.edu.cn

**Table S1. The sequences of primers and oligonucleotides used in this study**

| **Primers** | |
| --- | --- |
| circ-XRN2 F | TTCACATCTGATGGCTCCCC |
| circ-XRN2 R | TCCACTAGGAGTGAAAGCTGG |
| Linear XRN2 F | ATCCGAGACAAGCAGCCTATG |
| Linear XRN2 R | TCCTCTGGCTCAGGTTCACT |
| GAPDH F | GTCAAGGCTGAGAACGGGAA |
| GAPDH R | AAATGAGCCCCAGCCTTCTC |
| LCN2 F | TGCAGGTGGTACGTTGTGG |
| LCN2 R | TGTTGTCGTCCTTGAGGC |
| NRARP F | GTACCTTCCCGCCAACTACC |
| NRARP R | CTCGGGATTCCGAACTTGCT |
| KRT80 F | CCCCTACCCCTTATGTTCCCT |
| KRT80 R | GATATGGAGCGGAGTGGCTC |
| hsa_circ_0008260 F | ACTTTTGCTATGTTGGATGCAGT |
| hsa_circ_0008260 R | AAAAGAATTAAGACCGCTGCCC |
| hsa_circ_0057607 F | CTATGTAGCTTACAACGAATCTGAG |
| hsa_circ_0057607 R | TGAGACCCAGGTCTTAGGCA |
| hsa_circ_0126289 F | AAGGACCCAGTCCTCCCAAT |
| hsa_circ_0126289 R | ACTTGATGTTCTCTGCCATCTTCT |
| hsa_circ_0000079 F | ATTCATTTGAAGTGCTTTGCTGC |
| hsa_circ_0000079 R | CCTGGAGCTGTTCAACCGAA |
| hsa_circ_0101698 F | TAGAACTGAAGCATGCAAAGGA |
| hsa_circ_0101698 R | CGGCCTGTAGAACATCTTTATTTT |
| hsa_circ_0120885 F | GATAAAGCTGTTTCTCTCCAGCG |
| hsa_circ_0120885 R | TGTCCCTTATCAGCAGCTTCAA |
| hsa_circ_0006208 F | AGTCAACCTATGGAATCCAATCCC |
| hsa_circ_0006208 R | AGAGTGCCTGAAATGCTGGG |
| hsa_circ_0122120 F | GATTTGGAAACCAGGCCTACG |
| hsa_circ_0122120 R | GTTTCTTCTTGCTCCGTTCGT |
| hsa_circ_0007300 F | CCGTGGTACAGGGATGTGAC |
| hsa_circ_0007300 R | ACTCCAGCAACAAATGACCAGA |
| hsa_circ_0004999 F | CCTAGAGCTGCCAAGAAGCA |
| hsa_circ_0004999 R | ACCTTCTTTTCCATATGCTTTGAC |
| hsa_circ_0003958 F | CAGCACTATTGATCCTGCAGTT |
| hsa_circ_0003958 R | ACTGGTGGGCAGCATTGTAA |
| hsa_circ_0001380 F | ATTCCGGCCACCCATTGATT |
| hsa_circ_0001380 R | CAGGCCGTCGTCTTTTAGGA |
| hsa_circ_0137069 F | ACTGTGAAGCAAATTCGTCAG |
| hsa_circ_0137069 R | TCAGGACATTCCTTGCGCTT |
| **siRNAs Targeting sequence** | |
| Si circ-XRN2 1# | GAGGATAATGTCAGGACAGTT |
| Si circ-XRN2 2# | AATGTCAGGACAGTTTTAGAA |
| Si WWTR1 1# | TGGGTGTTAATTGAAATTTATAC |
| Si WWTR1 2# | CTGCGTTCTTGTGACAGATTATA |
| Si WWTR1 3# | TTGAGAGTAACTTAAGTTAAACA |
| Si YAP 1# | GAGATACTTCTTAAATCACATCG |
| Si YAP 2# | AGCAAATTCTCCAAAATGTCAGG |
| Si YAP 3# | GGCTAATACAGAAAAAGATGAAC |
| Si LATS1 | CTGGATCTATCAAATAAAGAAGT |
| Si LCN2 | CTCAAAACAGGGAGTACTTCAAG |
| Si LDHA | CAGGGATATTATTGACTAATAGC |
| Si LDHB | GGCAACAGTTCCAAACAATAAGA |
| **Probes for RNA Fluorescence in situ hybridization** | |
| Hsa_circ_0001134-CY3 | AACTGTCCTGACATTATCCTC |
