# Supplementary material for: CircXRN2 suppresses tumor progression driven by histone lactylation through activating the Hippo pathway in human bladder cancer
Source: Mol Cancer. 2023 Sep 8;22:151. doi: 10.1186/s12943-023-01856-1 (PMC10486081; doi:10.1186/s12943-023-01856-1)

Figure S1. **CircXRN2 activates the Hippo signaling pathway to suppress tumorigenesis**

**a.** CircXRN2 increased the expression level of LATS1 and caused corresponding alterations in TAZ and YAP. **b.** Overexpression of circXRN2 led to cytoplasmic retention of TAZ/YAP. Scale bar: 50 μm. **c.** CCK-8 assay proved that the Hippo pathway was vital for cell viability. **d.** Deficiency of TAZ/YAP inhibited colony formation in T24 and EJ cells. **e.** The cell apoptosis rate was investigated by flow cytometry with Annexin V-FITC and PI staining. **f.** The TAZ/YAP-deficient group had fewer migrated cells than the control group in the Transwell migration assay. **g.** Wound healing assay showed that TAZ/YAP promoted cell migration. All the data are presented as the mean ± standard deviation (n=3). *P <0.05, **P<0.01, compared with the control group.

Figure S1


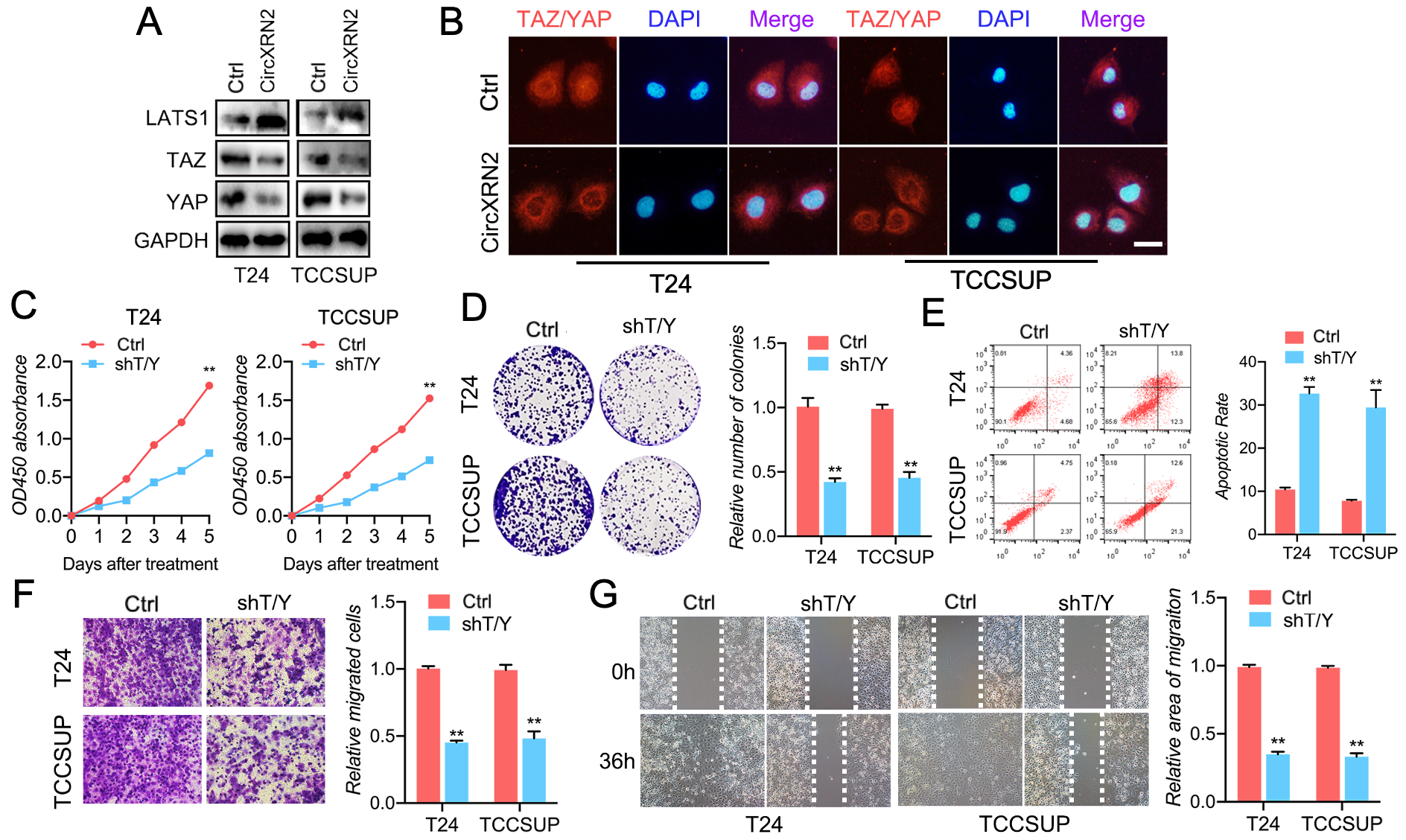

Supplement: Supplementary file 8 — Additional file 8: Figure S1. CircXRN2 activates the Hippo signaling pathway to suppress tumorigenesis. a. CircXRN2 increased the expression level of LATS1 and caused corresponding alterations in TAZ and YAP. b. Overexpression of circXRN2 led to cytoplasmic retention of TAZ/YAP. Scale bar: 50 μm. c. CCK-8 assay proved that the Hippo pathway was vital for cell viability. d. Deficiency of TAZ/YAP inhibited colony formation in T24 and EJ cells. e. The cell apoptosis rate was investigated by flow cytometry with Annexin V-FITC and PI staining. f. The TAZ/YAP-deficient group had fewer migrated cells than the control group in the Transwell migration assay. g. Wound healing assay showed that TAZ/YAP promoted cell migration. All the data are presented as the mean ± standard deviation (n=3). *P <0.05, **P<0.01, compared with the control group. [file 12943_2023_1856_MOESM8_ESM.docx]
