# Supplementary material for: CircXRN2 suppresses tumor progression driven by histone lactylation through activating the Hippo pathway in human bladder cancer
Source: Mol Cancer. 2023 Sep 8;22:151. doi: 10.1186/s12943-023-01856-1 (PMC10486081; doi:10.1186/s12943-023-01856-1)

Figure S2. **The Hippo pathway modulates glycolysis in human bladder cancer**

**a.** Glucose uptake was detected by flow cytometry in cells incubated with 2-NBDG. **b.** Depletion of TAZ/YAP abolished glucose uptake in T24 and TCCSUP cells. **c.** Impairment of the Hippo pathway reduced lactate production. **d.** The glycolytic rate in TAZ/YAP knockdown tumor cells was remarkably lower than that in normal tumor cells, as determined by a Seahorse metabolic analyzer. All the data are presented as the mean ± standard deviation (n=3). *P <0.05, **P<0.01.

Figure S2


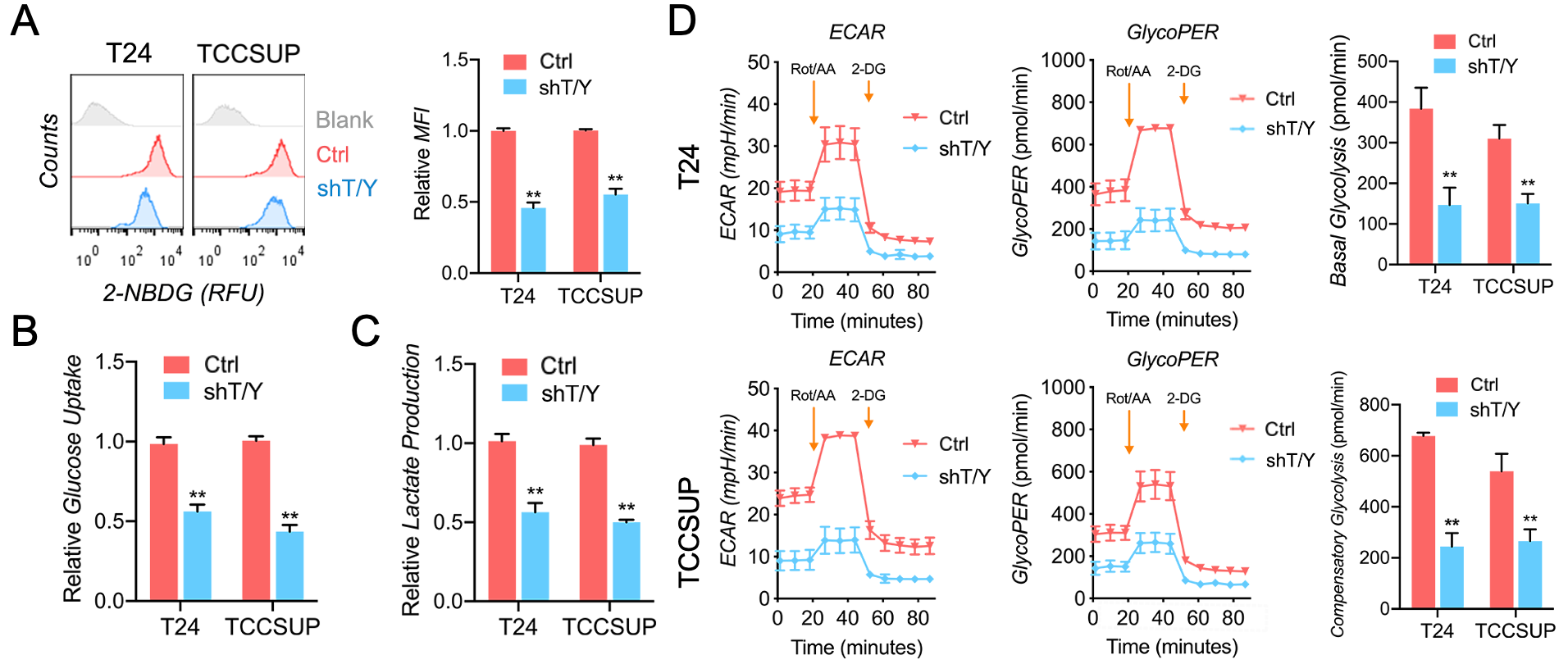

Supplement: Supplementary file 9 — Additional file 9: Figure S2. The Hippo pathway modulates glycolysis in human bladder cancer. a. Glucose uptake was detected by flow cytometry in cells incubated with 2-NBDG. b. Depletion of TAZ/YAP abolished glucose uptake in T24 and TCCSUP cells. c. Impairment of the Hippo pathway reduced lactate production. d. The glycolytic rate in TAZ/YAP knockdown tumor cells was remarkably lower than that in normal tumor cells, as determined by a Seahorse metabolic analyzer. All the data are presented as the mean ± standard deviation (n=3). *P <0.05, **P<0.01. [file 12943_2023_1856_MOESM9_ESM.docx]
