# Supplementary material for: CircXRN2 suppresses tumor progression driven by histone lactylation through activating the Hippo pathway in human bladder cancer
Source: Mol Cancer. 2023 Sep 8;22:151. doi: 10.1186/s12943-023-01856-1 (PMC10486081; doi:10.1186/s12943-023-01856-1)

Figure S3. **LCN2 attenuates antitumor effects induced by glycolysis inhibition**

**a.** Cell viability was measured in LDH-deficient cells with or without overexpression of LCN2. **b.** Colony formation assay indicated the rescue effect of LCN2 on LDH silencing. **c-d.** Transwell migration assays and wound healing assays were performed in LDH-deficient cells with or without overexpression of LCN2. All the data are presented as the mean ± standard deviation (n=3). *P <0.05, **P<0.01, compared with the control group.

Figure S3


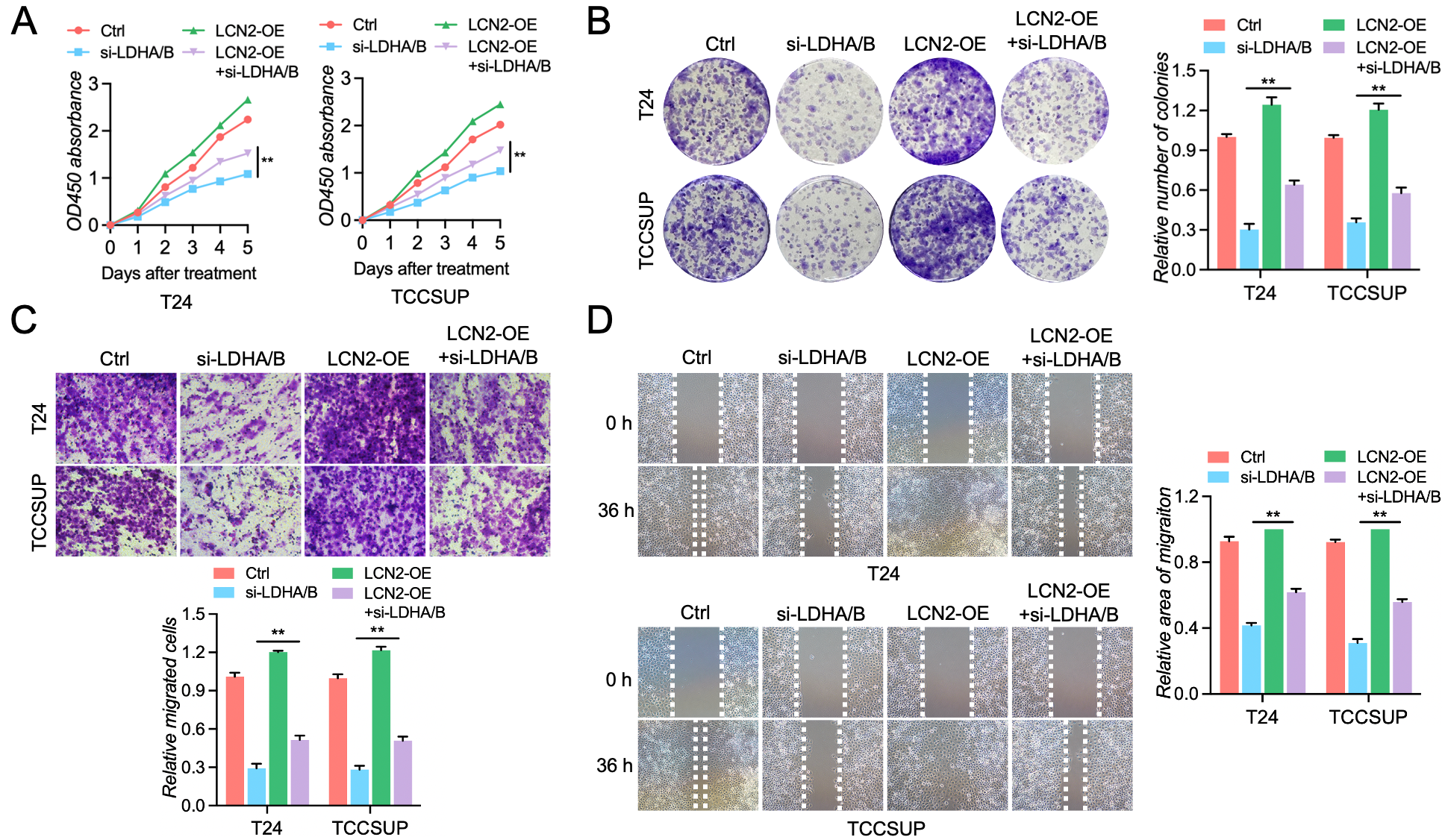

Supplement: Supplementary file 10 — Additional file 10: Figure S3. LCN2 attenuates antitumor effects induced by glycolysis inhibition. a. Cell viability was measured in LDH-deficient cells with or without overexpression of LCN2. b. Colony formation assay indicated the rescue effect of LCN2 on LDH silencing. c-d. Transwell migration assays and wound healing assays were performed in LDH-deficient cells with or without overexpression of LCN2. All the data are presented as the mean ± standard deviation (n=3). *P <0.05, **P<0.01, compared with the control group. [file 12943_2023_1856_MOESM10_ESM.docx]
