# Supplementary material for: CircXRN2 suppresses tumor progression driven by histone lactylation through activating the Hippo pathway in human bladder cancer
Source: Mol Cancer. 2023 Sep 8;22:151. doi: 10.1186/s12943-023-01856-1 (PMC10486081; doi:10.1186/s12943-023-01856-1)

Figure S4. **The knockdown efficiency of LDHA/B in cells after transfection with si-LDHA/B**

The expression levels of LDHA and LDHB were measured in LDH-deficient cells by western blotting to confirm the knockdown efficiency of LDHA and LDHB.

Figure S4


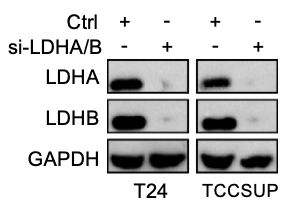

Supplement: Supplementary file 11 — Additional file 11: Figure S4. The knockdown efficiency of LDHA/B in cells after transfection with si-LDHA/B. The expression levels of LDHA and LDHB were measured in LDH-deficient cells by western blotting to confirm the knockdown efficiency of LDHA and LDHB. [file 12943_2023_1856_MOESM11_ESM.docx]
