# Supplementary material for: CircXRN2 suppresses tumor progression driven by histone lactylation through activating the Hippo pathway in human bladder cancer
Source: Mol Cancer. 2023 Sep 8;22:151. doi: 10.1186/s12943-023-01856-1 (PMC10486081; doi:10.1186/s12943-023-01856-1)

Figure S5. **The interaction between SPOP and LATS1 was verified in BCa cells**

**a.** We contrasted different fragments of the LATS1 protein to test the exact region interacting with circXRN2 in T24 and TCCSUP cells. **b.** Flag-LATS1-containing wild-type or mutant SBCs and HA-SPOP were transfected into BCa cells. Western blotting indicated that mutation of SBC1 led to remarkable blockade of LATS1 degradation mediated by SPOP, while depletion of SBC2 had little effect. **c.** Co-IP results showed that wild-type LATS1 could bind to SPOP, but the interaction of SBC1-mutant LATS1 with SPOP was almost completely diminished. Western blotting was performed to determine the expression levels of Flag-LATS1 and HA-SPOP.

Figure S5


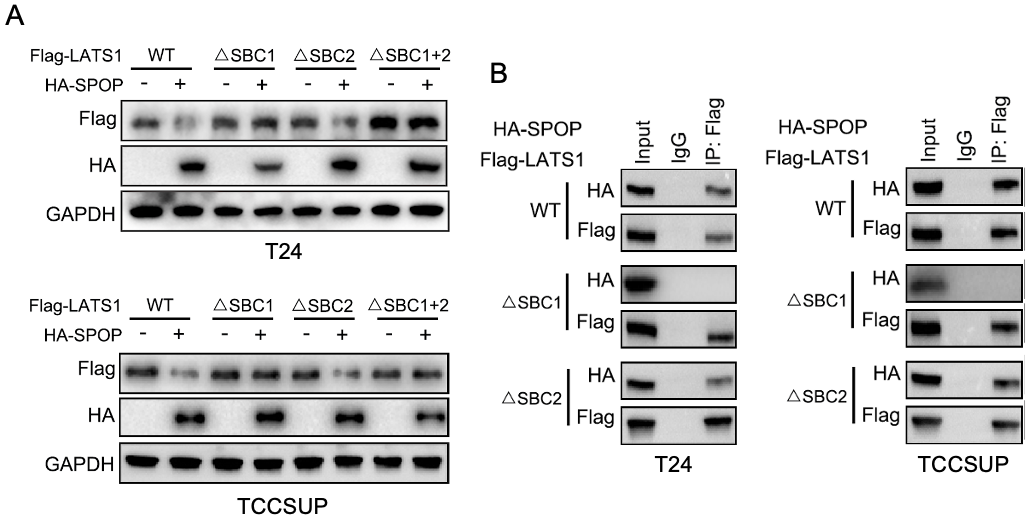

Supplement: Supplementary file 12 — Additional file 12: Figure S5. The interaction between SPOP and LATS1 was verified in BCa cells. a. We contrasted different fragments of the LATS1 protein to test the exact region interacting with circXRN2 in T24 and TCCSUP cells. b. Flag-LATS1-containing wild-type or mutant SBCs and HA-SPOP were transfected into BCa cells. Western blotting indicated that mutation of SBC1 led to remarkable blockade of LATS1 degradation mediated by SPOP, while depletion of SBC2 had little effect. c. Co-IP results showed that wild-type LATS1 could bind to SPOP, but the interaction of SBC1-mutant LATS1 with SPOP was almost completely diminished. Western blotting was performed to determine the expression levels of Flag-LATS1 and HA-SPOP. [file 12943_2023_1856_MOESM12_ESM.docx]
