# Supplementary material for: CircXRN2 suppresses tumor progression driven by histone lactylation through activating the Hippo pathway in human bladder cancer
Source: Mol Cancer. 2023 Sep 8;22:151. doi: 10.1186/s12943-023-01856-1 (PMC10486081; doi:10.1186/s12943-023-01856-1)

Figure S6. **The results of CUT&Tag with IgG in T24 cells**

**a.** The enrichment of DNA fragments in CUT&Tag with H3K18la or IgG. **b-d.** The enrichment peaks in the promoter regions of the LCN2, NRARP and KRT80 genes are indicated.

Figure S6
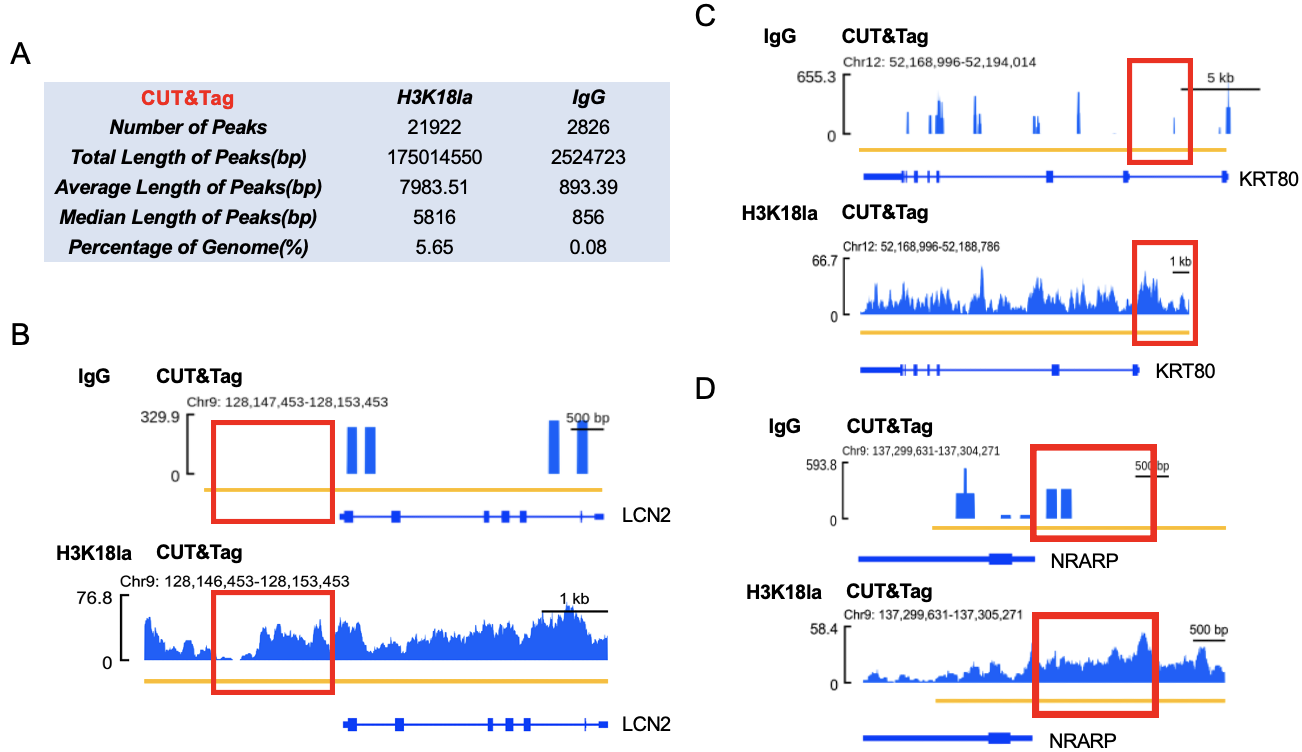

Supplement: Supplementary file 13 — Additional file 13: Figure S6. The results of CUT&Tag with IgG in T24 cells. a. The enrichment of DNA fragments in CUT&Tag with H3K18la or IgG. b-d. The enrichment peaks in the promoter regions of the LCN2, NRARP and KRT80 genes are indicated. [file 12943_2023_1856_MOESM13_ESM.docx]
