# Supplementary material for: CircXRN2 suppresses tumor progression driven by histone lactylation through activating the Hippo pathway in human bladder cancer
Source: Mol Cancer. 2023 Sep 8;22:151. doi: 10.1186/s12943-023-01856-1 (PMC10486081; doi:10.1186/s12943-023-01856-1)

Figure S7. **Validation of candidate genes regulated by circXRN2**

LCN2, NRARP and KRT80 mRNA levels were measured in circXRN2-overexpressing cells.

Figure S7


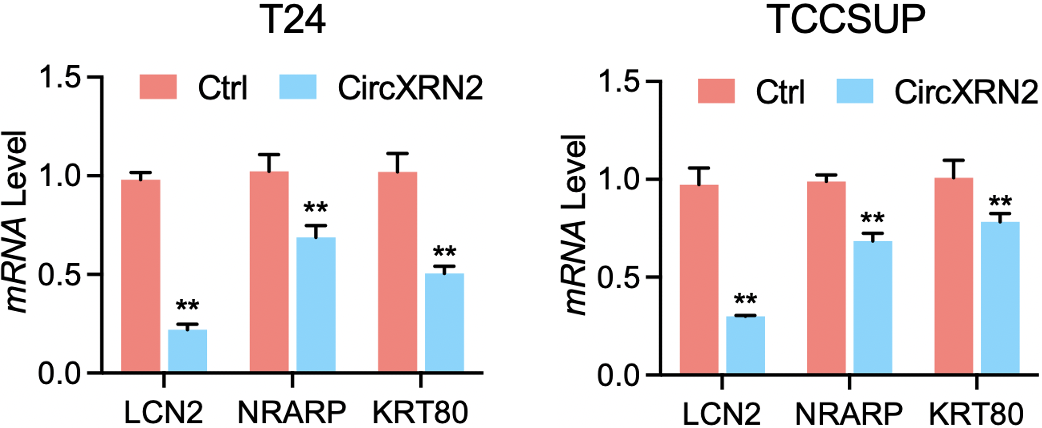

Supplement: Supplementary file 14 — Additional file 14: Figure S7. Validation of candidate genes regulated by circXRN2. LCN2, NRARP and KRT80 mRNA levels were measured in circXRN2-overexpressing cells. [file 12943_2023_1856_MOESM14_ESM.docx]
