# Supplementary material for: CircXRN2 suppresses tumor progression driven by histone lactylation through activating the Hippo pathway in human bladder cancer
Source: Mol Cancer. 2023 Sep 8;22:151. doi: 10.1186/s12943-023-01856-1 (PMC10486081; doi:10.1186/s12943-023-01856-1)

Figure S8. **The results of ChIP-qPCR with H3K18 in circXRN2-overexpressing cells**

ChIP assay following qPCR was used to detect the binding status of H3K18la in the LCN2 promoter region in circXRN2-overexpressing cells.

Figure S8


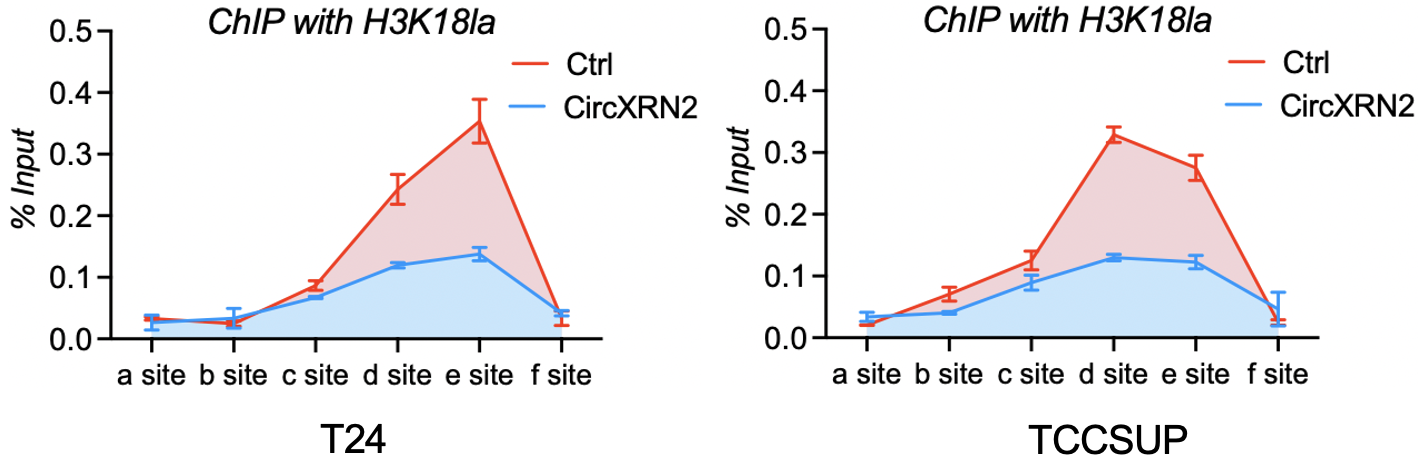

Supplement: Supplementary file 15 — Additional file 15: Figure S8. The results of ChIP-qPCR with H3K18 in circXRN2-overexpressing cells. ChIP assay following qPCR was used to detect the binding status of H3K18la in the LCN2 promoter region in circXRN2-overexpressing cells. [file 12943_2023_1856_MOESM15_ESM.docx]
